# Thermodynamics and H_2_ Transfer in a Methanogenic, Syntrophic Community

**DOI:** 10.1371/journal.pcbi.1004364

**Published:** 2015-07-06

**Authors:** Joshua J. Hamilton, Montserrat Calixto Contreras, Jennifer L. Reed

**Affiliations:** Department of Chemical and Biological Engineering, University of Wisconsin-Madison, Madison, Wisconsin, United States of America; The Pennsylvania State University, UNITED STATES

## Abstract

Microorganisms in nature do not exist in isolation but rather interact with other species in their environment. Some microbes interact via syntrophic associations, in which the metabolic by-products of one species serve as nutrients for another. These associations sustain a variety of natural communities, including those involved in methanogenesis. In anaerobic syntrophic communities, energy is transferred from one species to another, either through direct contact and exchange of electrons, or through small molecule diffusion. Thermodynamics plays an important role in governing these interactions, as the oxidation reactions carried out by the first community member are only possible because degradation products are consumed by the second community member. This work presents the development and analysis of genome-scale network reconstructions of the bacterium *Syntrophobacter fumaroxidans* and the methanogenic archaeon *Methanospirillum hungatei*. The models were used to verify proposed mechanisms of ATP production within each species. We then identified additional constraints and the cellular objective function required to match experimental observations. The thermodynamic *S*. *fumaroxidans* model could not explain why *S*. *fumaroxidans* does not produce H_2_ in monoculture, indicating that current methods might not adequately estimate the thermodynamics, or that other cellular processes (e.g., regulation) play a role. We also developed a thermodynamic coculture model of the association between the organisms. The coculture model correctly predicted the exchange of both H_2_ and formate between the two species and suggested conditions under which H_2_ and formate produced by *S*. *fumaroxidans* would be fully consumed by *M*. *hungatei*.

## Author Summary

Natural and engineered microbial communities can contain up to hundreds of interacting microbes. These interactions may be positive, negative, or neutral, as well as obligate or facultative. Syntrophy is an obligate, positive interaction, in which one species lives off the metabolic by-products of another. Syntrophic associations play an important role in sustaining a variety of natural communities, including those involved in the breakdown and conversion of short-chain fatty acids (e.g., propionate) to methane. In many syntrophic communities, electrons are transferred from one species to the other through small molecule diffusion. In this work, we expand the study of a two-member syntrophic, methanogenic community through the development and analysis of computational models for both species: the bacterium *Syntrophobacter fumaroxidans* and the methanogenic archaeon *Methanospirillum hungatei*. These models were used to analyze energy conservation mechanisms within each species, as well as small molecule exchange between the two organisms in coculture. The coculture model correctly predicted the exchange of both H_2_ and formate between the two species and suggested conditions under which these molecules would be fully metabolized within the community.

## Introduction

Microorganisms in nature engage in a variety of interactions with other species in their environment. Syntrophy is one such type of inter-species interaction in which one species lives off the metabolic by-products of another [1–3]. Synthetic methanogenic communities [4] are typically tightly constrained by thermodynamics, as the oxidation reactions carried out by the first community member are thermodynamically unfavorable unless the degradation products are maintained at low levels by the second community member [5].

In anaerobic syntrophic communities, electrons are transferred from one partner to the other through direct contact or small molecule diffusion [6]. Traditional biochemistry has elucidated intracellular electron transport mechanisms [3,7–9], but it is difficult to evaluate these pathways in their metabolic and environmental context. Genome-scale metabolic models (GEMs) [10–12] and constraint-based methods are powerful computational tools for understanding individual pathways in a broader metabolic context including both isolated microbial species [13–15] and simple microbial communities [16–26].

One of the earliest microbial community models used flux balance analysis (FBA, [27]) to investigate formate and H_2_ exchange between the sulfate-reducing bacterium *Desulfovibrio vulgaris* and the methanogenic archaeon *Methanococcus maripaludis* [16]. In this study, each organism was modeled as a compartment within a larger community-scale model. Compartmentalized approaches have been used to study the origins of cooperation and competition [17–19], as well as specific communities [20–24]. These approaches [16–24] have often used a single (joint) objective function to capture community behavior. OptCom [25,26] instead uses a multi-level optimization framework, to capture the trade-offs between individual and community fitness, with separate objective functions for the individual species and the community. In addition, community FBA (cFBA) [28] extends compartmentalized approaches [16–24] to specifically account for individual species’ biomass abundance.

Genome-scale models can also be used to study the relationship between thermodynamics and metabolism, by ensuring that network predictions are consistent with thermodynamic principles [29–34]. In this study, we used thermodynamics-based metabolic flux analysis (TMFA) to develop a thermodynamic, coculture model of the syntrophic association between the anaerobic bacterium *Syntrophobacter fumaroxidans* and the methanogenic archaeon *Methanospirillum hungatei*. In association with *M*. *hungatei*, *S*. *fumaroxidans* converts propionate to acetate, CO_2_, and H_2_ [35–37]. CO_2_ and H_2_ can be interconverted to formate [38–40], with H_2_ and formate serving as the electron carriers between the two species. H_2_ and formate production are only observed during syntrophic growth. Using a thermodynamic, constraint-based model, we set out to test the proposed hypothesis that this behavior is governed by thermodynamics [3,5,6,8,9].

We developed genome-scale metabolic reconstructions of both microorganisms, and verified proposed mechanisms of ATP production within each individual species. Additional constraints and a cellular objective function were identified to predict the proper flux through experimentally characterized carbon and electron transport pathways during monoculture and syntrophic (i.e., coculture) growth. Our analysis revealed that thermodynamic constraints alone are insufficient to explain why *S*. *fumaroxidans* does not produce H_2_ in monoculture.

We also extended TMFA to model the syntrophic association between the two microorganisms. The association is modeled as a continuous coculture system with constraint-based models for each microbe and a mass balance around the reactor. Similar to cFBA [28], the coculture model accounted for the biomass concentrations of each species. We predicted the behavior of this syntrophic association under a variety of dilution rates, and identified regimes of behavior consistent with experimental observations.

## Results

Metabolic reconstructions of *S*. *fumaroxidans* (*i*Sfu648) and *M*. *hungatei* (*i*Mhu428) were tested and parameterized using experimental data from growth on single substrates in monoculture and coculture. The *i*Sfu648 and *i*Mhu428 models were used to verify proposed mechanisms of energy conservation within each species. A coculture model was then developed to identify conditions where H_2_ and formate, produced by *S*. *fumaroxidans*, is fully metabolized by *M*. *hungatei*.

### Testing and Parameterizing the *i*Mhu428 Metabolic Model

The *i*Mhu428 reconstruction of *M*. *hungatei* was built from the *i*MB745 reconstruction of *M*. *acetivorans* [41]. A preliminary draft reconstruction was built from *i*MB745 using the RAVEN Toolbox [42] and the KEGG SSDB [43]; however, *M*. *hungatei* orthologs were found for only 428 of the 745 genes in *i*MB745. To avoid extensive gapfilling, reactions from the *i*MB745 were copied into the *M*. *hungatei* reconstruction, with modifications to reflect key metabolic features of *M*. *hungatei* (see S1 Text). As a consequence, the *i*Mhu428 reconstruction is a draft reconstruction requiring further evaluation. A thermodynamic model for *i*Mhu428 was built and TMFA was used to predict ATP generating mechanisms in minimal media monoculture conditions (see S1 Table in S1 Dataset for constraints used).

**Fig 1:**
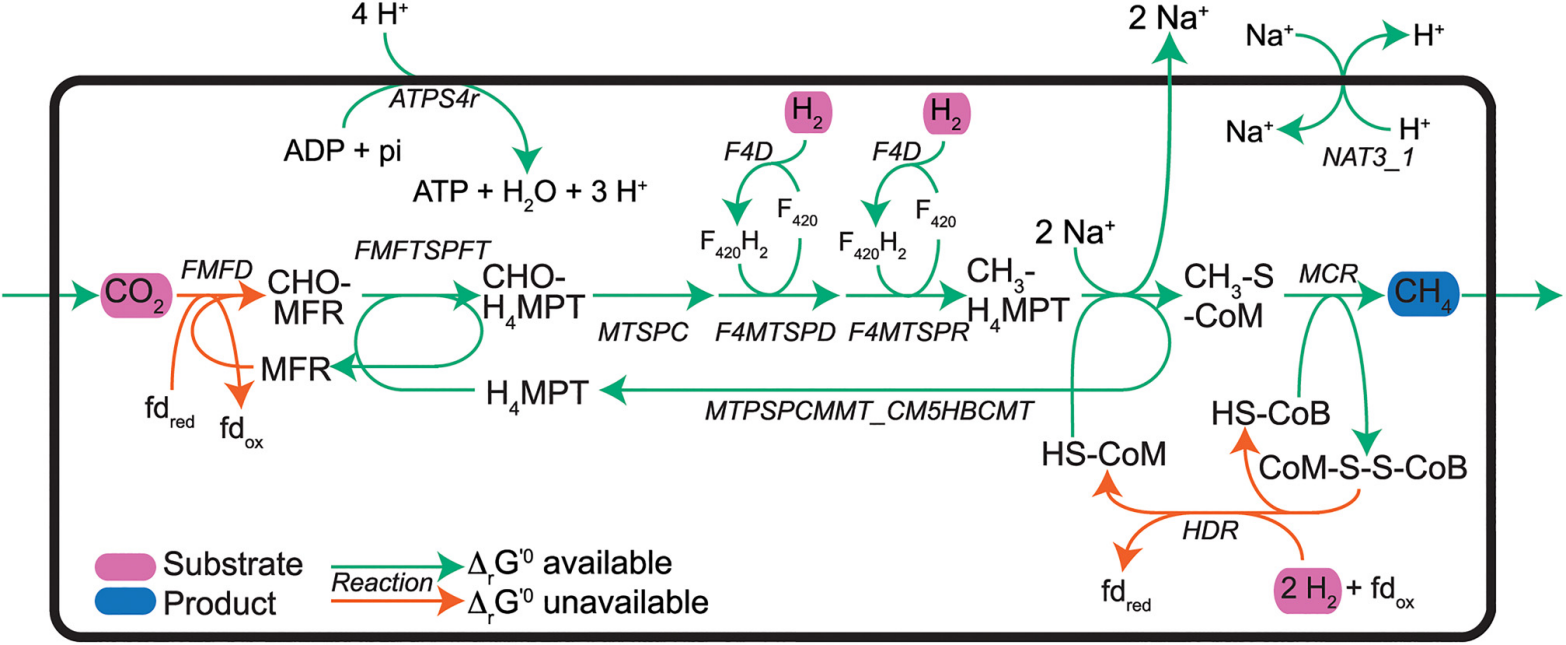
Carbon and electron transport pathways in *M*. *hungatei*. The methanogenesis pathway has an overall stoichiometry of CO_2_ + 4 H_2_ → CH_4_ + 2 H_2_O. Substrates and products are indicated with purple and blue ovals, respectively, while reactions with and without estimates are indicated with green and orange arrows, respectively. Metabolite and reaction abbreviations are given in S2 Dataset.

Experimental evidence suggests that *M*. *hungatei* is able to generate 0.5 mole ATP per mole of CO_2_ converted to CH_4_ [7], via the metabolic route shown in Fig 1. In order for this route to be thermodynamically feasible, the Δ_*r*_*G*^'0^ of one reaction (*FMFTSPFT*, formylmethanofuran-tetrahydromethanopterin formyltransferase) had to be allowed to vary within a 99% confidence interval of its estimated standard transformed Gibbs free energy of reaction ($\Delta_{r}G_{est}^{'0}$) (rather than the 95% interval used for all other reactions) in order to carry flux in the proper direction. The reactions for carbon source utilization in *M*. *hungatei* are well-characterized [7,44–47], but uncertainty remains about the stoichiometry of small ion transport [7]. Na^+^ transport stoichiometries associated with tetrahydromethanopterin S-methyltransferase (*MTSPCMMT_CM5HBCMT*, E.C. 2.1.1.86) and a Na^+^/H^+^ antiporter (*NAT3_1*) were selected to give an ATP yield matching the experimental estimates: two Na^+^ ions exported by *MTSPCMMT_CM5HBCMT*, and a one Na^+^ per H^+^ transported by *NAT3_1*. However, different stoichiometries for these reactions are also thermodynamically possible (see Discussion).

Experimental measurements of growth rates, yields, and maintenance costs were also used to identify substrate uptake rates (SUR) for CO_2_ and formate, and the growth- (GAM) and non-growth-associated (NGAM) ATP maintenance requirements for *M*. *hungatei*, as described in S1 Text. NGAM represents the amount of energy spent to maintain the cell (i.e., maintenance energy), while GAM represents energy spent on growth-related functions (e.g., protein synthesis). For the *i*Mhu428 model, the NGAM was estimated to be 0.6 mmol ATP/gDW/day, GAM was estimated to be 47 mmol ATP/gDW, SUR_CO2_ was estimated to be 75.7 mmol/gDW/day, and SUR_formate_ was estimated to be 955 mmol/gDW/day.

### Testing and Parameterizing the *i*Sfu648 Metabolic Model

The *i*Sfu648 reconstruction of *S*. *fumaroxidans* was built from the KEGG database using the RAVEN Toolbox [42]. The resulting draft reconstruction was manually refined (see S1 Text), with particular attention paid to ATP production mechanisms. A number of studies have identified gene clusters encoding a variety of hydrogenases, dehydrogenases, and other electron transport enzymes [8,9,48–51], whose expression levels vary across growth conditions [51]. All told, 17 enzymes which catalyze 12 different electron transport reactions have been identified (S3 Table in S1 Dataset). In many cases, the proposed reactions catalyzed by these enzymes differ between studies; a brief description of each reaction and justification for each annotation is given in S1 Text. The draft reconstruction was updated to be consistent with the reported carbon utilization and electron transport reactions, and the resulting stoichiometric model was converted to a thermodynamic model.

### ATP Production Mechanisms in *S*. *fumaroxidans*

Experimental studies have elucidated five growth modes for *S*. *fumaroxidans*: four in monoculture and one in coculture with *M*. *hungatei* (S1 Text) [36,48,52]. This work examines the three most commonly studied growth modes (Table 1): monoculture growth on fumarate, monoculture growth on fumarate plus propionate, and coculture growth on propionate.

**Table 1:** Experimentally observed and computationally predicted extracellular flux distributions for *S*. *fumaroxidans* examined in this study.

Growth Mode*	Experimental Stoichiometry (No Growth)^#^	Predicted Stoichiometry (Max Growth)	Observed Growth (1/days)	Predicted Growth (1/days)
Fumarate, Monoculture	7 fumarate → 6 succinate + 4 CO_2_	7 fumarate → 5.11 succinate + 4.75 CO_2_ + 0.25 acetate	0.33	0.33
Propionate + Fumarate, Monoculture	propionate + 3 fumarate → acetate + CO_2_ + 3 succinate	propionate + 3 fumarate → 0.59 acetate +1.25 CO2 + 2.83 succinate	0.73	0.41^$^
Propionate, Coculture	propionate → acetate + CO2 + 3 H_2_	propionate—> 0.99 acetate + 0.86 CO_2_ + 2.69 H_2_	0.22	0.22

Experimental measurements from fumarate-only and propionate-only conditions were used to parameterize the model.

*Monoculture simulations were performed with qualitative reaction direction constraints which prevent H_2_ production.

^#^Experimental stoichiometries were calculated from experimental measurements assuming no biomass growth [36,52].

^$^This growth rate was predicted after the model was modified to include an explicit constraint on the propionate to fumarate uptake ratio.

**Fig 2:**
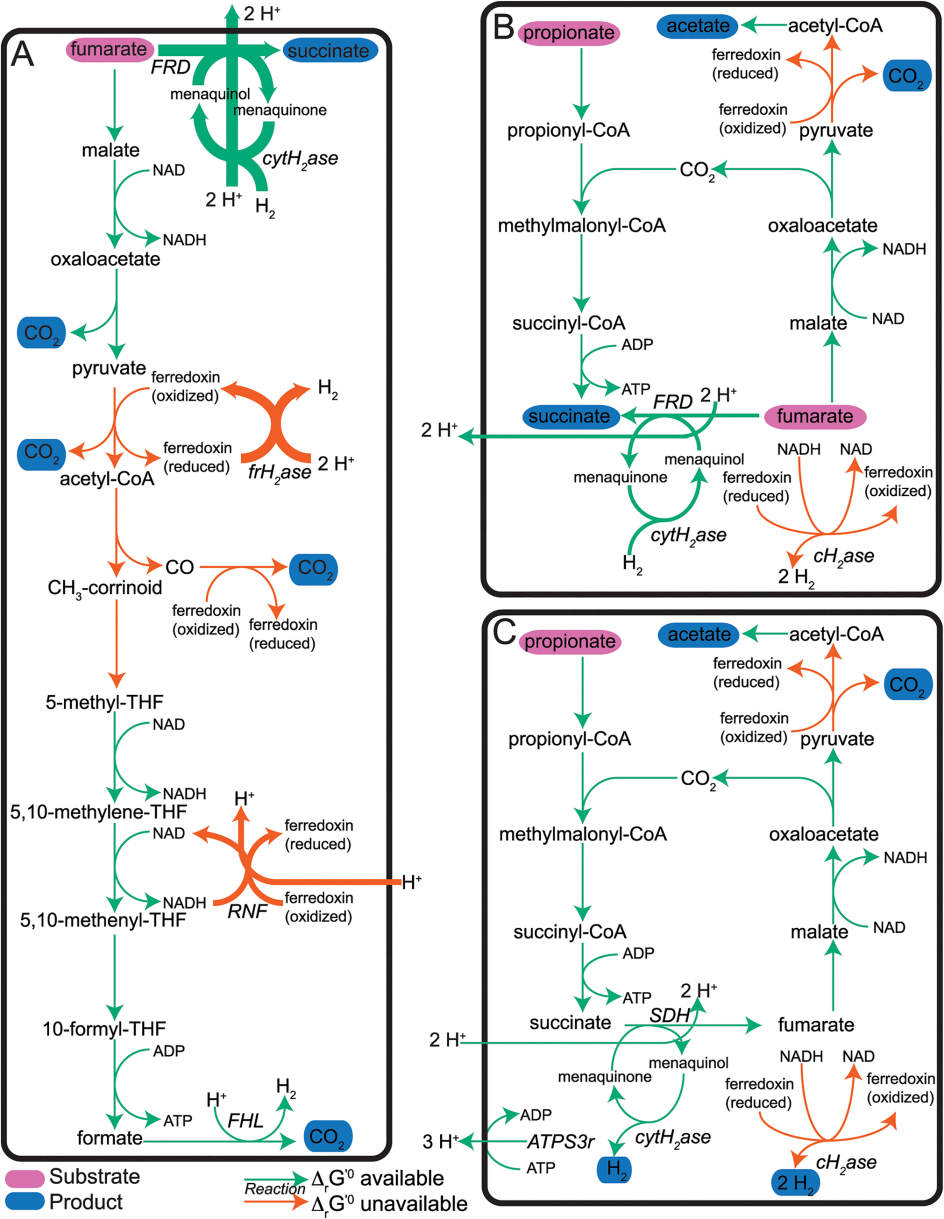
Carbon and electron transport pathways in *S*. *fumaroxidans* under different substrate conditions. Simulations maximized ATP production with no minimum biomass requirement. The overall stoichiometry of each pathway corresponds to the experimental stoichiometry given in Table 1. (A) Monoculture growth on fumarate alone. (B) Monoculture growth on fumarate and propionate. (C) Syntrophic growth on propionate. Substrates and products are indicated with purple and blue ovals, respectively, while reactions with and without estimates are indicated with green and orange arrows, respectively. Arrow thickness indicates relative flux values. Metabolite and reaction abbreviations are given in S4 Dataset.

A variety of experimental findings were synthesized to develop theoretical flux distributions for these three growth modes (Fig 2 and S1 Text). These experimental findings suggested additional regulatory and flux-coupling constraints for the *i*Sfu648 model, such as coupling between fumarate reductase and the cytosolic hydrogenase due to co-localization in the membrane (see S2 Table in S1 Dataset and S1 Text for the full set of constraints and their justification).

During monoculture growth on fumarate alone (Fig 2A), one mole of fumarate gets fully oxidized to CO_2_, while six moles of fumarate get reduced to succinate [48,52]:

$$7\;\text{fumarate}\to 6\;\text{succinate}\;+\;4\;{\text{CO}}_{2}$$ (1)

The oxidation of one fumarate to CO_2_ generates one ATP and five reducing equivalents (three NADH and two pairs of reduced ferredoxin) [48,52], while the reduction of additional fumarate to succinate by fumarate reductase (*FRD*) consumes reducing equivalents (menaquinol) [48]. Electrons are transferred from NADH and reduced ferredoxin to menaquinone through the combined action of the Rnf complex (*RNF*), the ferredoxin-oxidizing hydrogenase (*frH*_*2*_*ase*), the cytosolic hydrogenase (*cytH*_*2*_*ase*) and formate hydrogen lyase (*FHL*). The reduction of fumarate to succinate also generates the proton motive force (PMF) responsible for driving the *RNF* reaction and producing ATP.

During monoculture growth on fumarate plus propionate (Fig 2B), one mole of propionate gets oxidized to succinate, while one mole of fumarate gets oxidized to acetate and CO_2_. Two additional moles of fumarate get reduced to succinate [36,48]:

$$\text{propionate}\;+\;3\;\text{fumarate}\to \text{acetate}\;+\;{\text{CO}}_{2}\;+\;3\;\text{succinate}$$ (2)

The oxidation of fumarate to acetate and CO_2_ produces one NADH and one pair of reduced ferredoxin, while the reduction of fumarate to succinate by *FRD* consumes menaquinol. Electrons are transferred from NADH and reduced ferredoxin to menaquinone through the combined action of the confurcating hydrogenase (*cH*_*2*_*ase*) and *cytH*_*2*_*ase*. Oxidation of propionate to succinate produces one ATP, while *FRD* generates the PMF necessary for additional ATP production.

During coculture growth on propionate (Fig 2C), propionate gets oxidized to acetate and CO_2_ via the methylmalonyl-CoA pathway [48,52]:

$$\text{propionate}\to \text{acetate}\;+\;{\text{CO}}_{2}\;+\;3\;{\text{H}}_{2}$$ (3)

ATP is generated during the oxidation of propionate to succinate, and this ATP establishes the PMF necessary to drive the endergonic oxidation of succinate to fumarate (*SDH*), producing menaquinone. *cytH*_*2*_*ase* then transfers electrons from menaquinol to two protons, generating H_2_. The oxidation of fumarate to acetate and CO_2_ produces one NADH and one pair of reduced ferredoxin, and *cH*_*2*_*ase* couples NADH and ferredoxin re-oxidation with H_2_ production. Unlike in the monoculture growth modes, the H_2_ is not consumed intracellularly and must diffuse outside the cell. It has been proposed that the net production of H_2_ by *S*. *fumaroxidans* is only thermodynamically favorable at the low H_2_ concentrations maintained by methanogens, thereby explaining why *S*. *fumaroxidans* only produces H_2_ during coculture growth.

**Fig 3:**
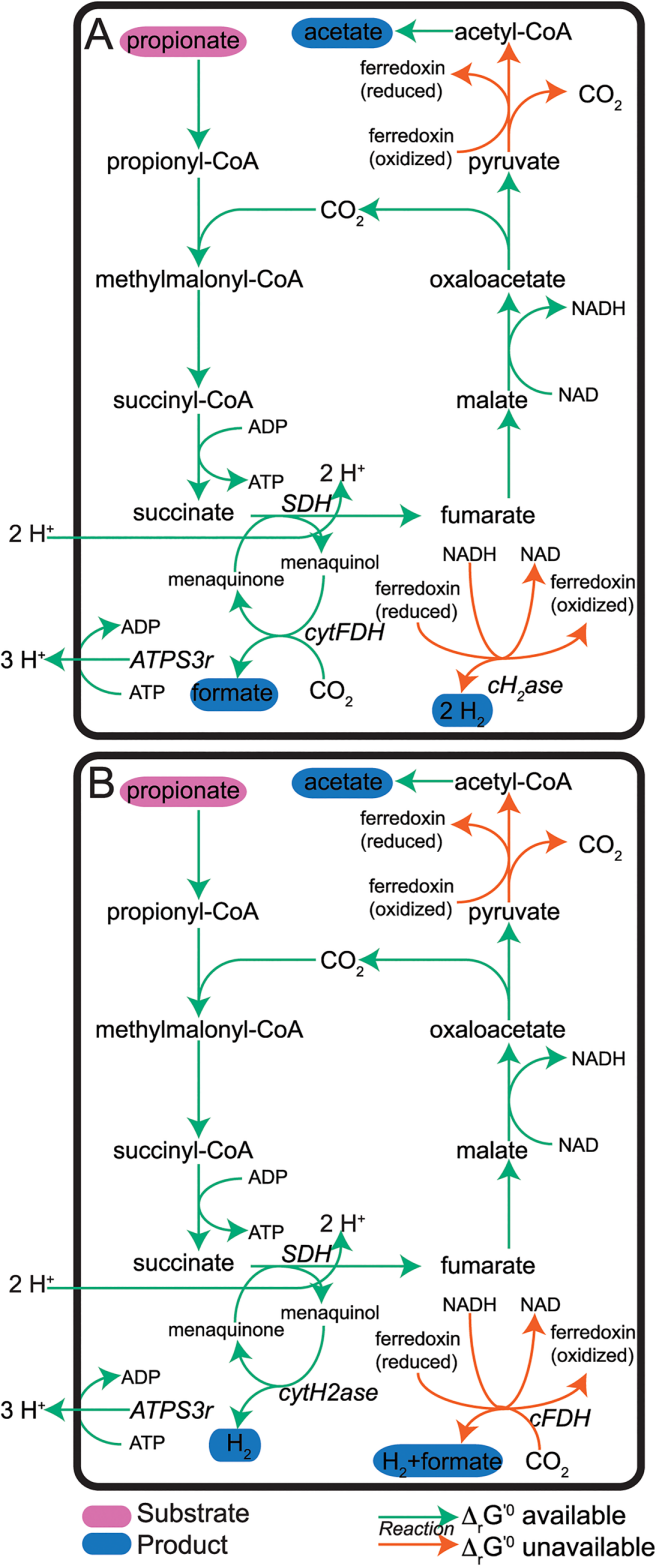
Alternative carbon and electron transport pathways in *S*. *fumaroxidans* under coculture growth conditions. (A) Formate production via *cytFDH*. (B) Formate production via *cFDH*. (A and B) Simulations maximized ATP production with no minimum biomass requirement. The overall stoichiometry of each pathway corresponds to the alternative stoichiometry given in Eq 4. Substrates and products are indicated with purple and blue ovals, respectively, while reactions with and without estimates are indicated with green and orange arrows, respectively. Arrow thickness indicates relative flux values. Metabolite and reaction abbreviations are given in S4 Dataset.

*S*. *fumaroxidans* exhibits considerable flexibility in its ATP production mechanisms during coculture growth ([8,9,48,51]), and can produce formate instead of CO_2_ (Fig 3) yielding an overall transformation of:

$$\text{propionate}\to \text{acetate}\;+\;\text{formate}\;+\;2\;{\text{H}}_{2}$$ (4)

In one mechanism (Fig 3A), activity of the cytosolic formate hydrogenase (*cytFDH*) substitutes for the activity of *cytH*_*2*_*ase*. In a second mechanism (Fig 3B), the confurcating formate dehydrogenase (*cFDH*) substitutes for *cH*_*2*_*ase*. Here, *cFDH* couples NADH and ferredoxin re-oxidation with the conversion of CO_2_ (from propionate oxidation) to formate.

### Model Predictions of ATP Production by *S*. *fumaroxidans*

Experimental evidence and conceptual models of *S*. *fumaroxidans* energy metabolism suggest that the carbon and electron transfer pathways shown in Fig 2 provide the sole source for ATP production in *S*. *fumaroxidans*, either by substrate-level phosphorylation or through establishment of a proton gradient used by ATP synthase [5]. To test the computational model’s predictions, TMFA was used to maximize ATP production under each of the three growth modes.

When flux was restricted to a reduced network containing all the reactions shown in Fig 2 (listed in S4 Table in S1 Dataset), the *i*Sfu648 model correctly predicted the flux distributions shown in Fig 2. However, when flux was allowed throughout the entire network, additional flux distributions with higher ATP yields were identified. Additional reaction direction constraints were developed to ensure model-predicted flux distributions matched experimental observations (see S2 Table in S1 Dataset and S1 Text for details). However, the resulting flux distributions are not fully consistent with the hypothesis that *S*. *fumaroxidans* has adapted to maximize its energy yield.

### Evaluating Thermodynamics of H_2_ Production

Experimental studies of *S*. *fumaroxidans* have shown that H_2_ is not produced during growth in monoculture [36,52,53], and it is widely thought that H_2_ production is only thermodynamically favorable at low partial pressures [3,6,8,9]. In particular, methanogens in syntrophic communities enable sustained H_2_ production by consuming H_2_ and keeping its partial pressure low [3,5,6,8,9]. Indeed, when H_2_ production was observed in monoculture, H_2_ production ceased at a partial pressure of approximately 10 Pa [53].

However, when maximizing H_2_ production under monoculture conditions, simulations reveal that H_2_ production remains thermodynamically feasible. For example, during monoculture growth on fumarate, the *i*Sfu648 model predicts that H_2_ can be produced via the following mechanism:

$$\text{fumarate}\to 4\;{\text{CO}}_{2}+\;6\;{\text{H}}_{2}$$ (5)

In this scenario, H_2_ molecules produced by the ferredoxin-oxidizing hydrogenase are exported outside the cell, instead of serving as substrates for the cytosolic hydrogenase. As a result, no PMF is generated by fumarate reductase, and the net ATP yield is zero. Thus, while H_2_ production remains thermodynamically possible, H_2_ production is only associated with sub-optimal mechanisms of ATP generation. This suggests that thermodynamic considerations alone may not explain the absence of H_2_ production during monoculture growth, but that the observed flux distribution may instead be driven by demands for energy generation.

While H_2_ production was not predicted for monoculture conditions when ATP production was maximized, H_2_ production was initially predicted when growth was instead maximized. To eliminate monoculture H_2_ production in the TMFA model, we first sought to constrain ratios of metabolite concentrations with an approach similar to that used to correct TMFA growth predictions [34] (see S1 Text for details). While metabolite ratio constraints could be identified to prevent some H_2_ production mechanisms, H_2_ production during monoculture growth could not be completely eliminated. If thermodynamics prevents H_2_ production in monoculture, then the current thermodynamic model may contain too much uncertainty in its Gibbs free energy estimates (see Discussion). Regulatory effects could also potentially prevent H_2_ production in monoculture conditions. To correct the model, all subsequent monoculture simulations were performed by preventing H_2_ production.

### Parameterization of the *i*Sfu648 Metabolic Model

Model parameters were estimated after reaction direction constraints were added to the *i*Sfu648 model (to be consistent with reported ATP generation and H_2_ production mechanisms). Experimental measurements of growth rates, yields, and maintenance costs were used to identify the SURs, GAM, and NGAM parameters for *S*. *fumaroxidans*. These parameters were estimated using data from monoculture growth on fumarate alone and coculture growth on propionate alone (see S1 Text). For the *i*Sfu648 model, the following parameters resulted in the best fit of the model to the experimental data: NGAM = 3.36 mmol ATP/gDW/day, GAM = 22.8 mmol ATP/gDW, SUR_propionate_ = 37.7 mmol/gDW/day, and SUR_fumarate_ = 27.6 mmol/gDW/day.

Using these parameter values, the *in silico* growth rates under each growth condition were predicted (Table 1). Not surprisingly, the predicted growth rates for fumarate alone and propionate alone conditions agree with experimental observations (since these were used to estimate the parameter values). However, the model significantly under-predicts the measured growth rate during monoculture growth on fumarate plus propionate (0.55 days^-1^ predicted, 0.73 days^-1^ observed). This discrepancy could be caused by differences in uptake rates or maintenance costs in the fumarate plus propionate condition compared to the conditions with propionate alone or fumarate alone.

### Uptake and Secretion at Maximal Growth

When maximizing biomass production on the entire network, the *i*Sfu648 model predicted a wide range of product secretion rates. When the enzyme cost (i.e., total flux) was minimized at the maximum growth (pTMFA [54], see Methods) the model-predicted product yields closely matched reported values for two of the three growth modes (Table 1)—monoculture growth on fumarate alone and coculture growth on propionate alone. These results indicate that the majority of carbon is diverted to fermentation products, consistent with the expectation that high fluxes through the low-energy fermentation pathways are needed to meet cellular energy demands.

However, for monoculture growth on fumarate plus propionate, the model failed to predict that fumarate and propionate should be consumed at the observed ratio of approximately three fumarate per propionate [36]. Instead, the model predicted both substrates would be consumed at their maximum SURs, resulting in a ratio of 0.73 fumarate per propionate. The experimentally observed 3:1 ratio is thought to arise due to coupling within the metabolic network (Fig 2B), since oxidation of one fumarate produces one CO_2_ (used to oxidize one propionate) and two pairs of electrons (used to reduce two fumarate). While this coupling arises naturally on the reduced network, the full metabolic network enables alternative coupling mechanisms (not shown) that permit other fumarate to propionate ratios. Since propionate oxidation generates carbon precursors and ATP for biomass, maximizing biomass production results in the model under-predicting the fumarate to propionate uptake ratio. Increasing the ratio of fumarate to propionate uptake (to 3:1) decreases the predicted propionate SUR and growth rate (S1 Fig in S1 Text), implying the experimental ratio is sub-optimal with respect to growth maximization. Instead of constraining SURs (since values were not reported in the literature), we incorporated a fumarate to propionate SUR ratio constraint for this condition.

While the predicted product yields closely matched experimental observations (after imposing the SUR ratio constraint), the pTMFA-predicted intracellular flux distribution during monoculture growth on fumarate and propionate substantially deviated from that shown in Fig 2B. Further constraints on reaction directions were required so that propionate and fumarate were metabolized in the model via the pathways shown in Fig 2B (results not shown). Taken together, the need for constraints on reaction directions and fumarate to propionate uptake ratio suggests that neither maximization of biomass nor minimization of enzyme cost are sufficient to explain the fluxes of *S*. *fumaroxidans* growing in this monoculture condition.

### Behavior of *M*. *hungatei* and *S*. *fumaroxidans* in Coculture

During growth in coculture, *S*. *fumaroxidans* converts propionate to acetate, H_2_, and CO_2_ or formate [36,52], while *M*. *hungatei* consumes acetate, CO_2_, H_2_, and formate and produces CH_4_ [55]. *M*. *hungatei* can also optionally interconvert excess CO_2_ and H_2_ to formate via a formate dehydrogenase [56].

Cocultures of *M*. *hungatei* and *S*. *fumaroxidans* have been grown in both batch and continuous (chemostat) systems. During batch growth, H_2_ pressure rose during the lag phase and became constant during exponential growth [53]. Continuous cultures also exhibited constant H_2_ partial pressure [53]. However, to the best of our knowledge, measurements for the relative ratios of *M*. *hungatei* to *S*. *fumaroxidans* at constant H_2_ pressure (where H_2_ consumption and production rates are balanced) have not been reported. Instead, an overall reaction for the coculture of

$$\text{propionate}\to \text{acetate}\;+\;0.25\;{\text{CO}}_{2}+\;0.75\;{\text{CH}}_{4}$$ (6)

is frequently discussed [5,53], which can occur at a ratio of three *M*. *hungatei* to four *S*. *fumaroxidans*. Since initial conditions for batch experiments were not reported, a continuous culture model was constructed and first evaluated using a 3:4 relative biomass ratio (*M*. *hungatei*: *S*. *fumaroxidans)*.

The continuous coculture model included constraint-based models for each microbe and mass balances around the reactor (S2 Fig in S1 Text), and accounted for the biomass concentrations of each species. Both species were constrained to grow at the dilution rate, and the model minimized the species-weighted total flux through the two metabolic networks (pTMFA). Propionate was the only substrate in the reactor feed (i.e., it had a net flux into the reactor), ensuring that all carbon and electrons used by *M*. *hungatei* were produced by *S*. *fumaroxidans*.

**Fig 4:**
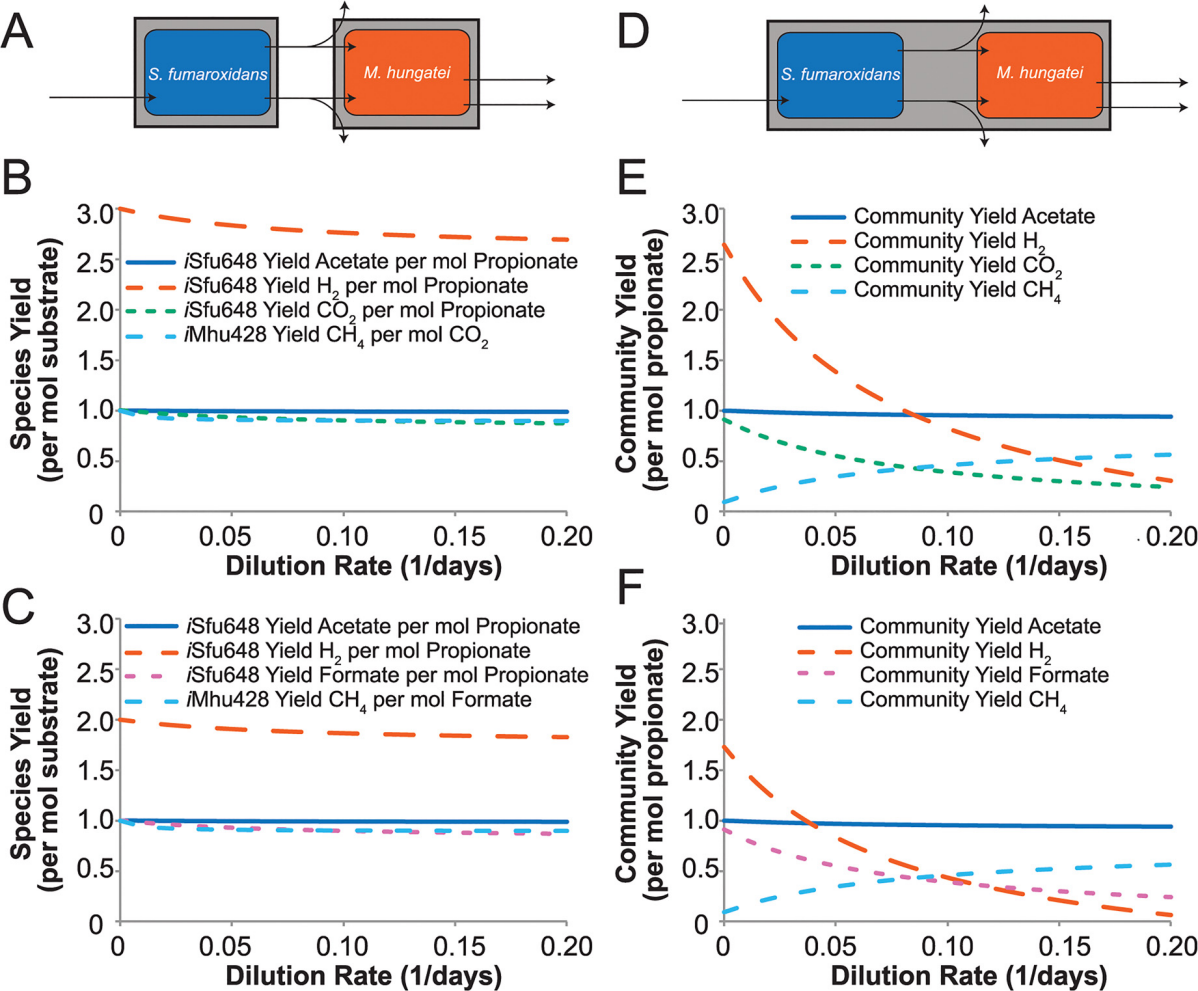
Predicted individual and community by-product yields in the coculture system at a ratio of three *M*. *hungatei* to four *S*. *fumaroxidans*. (A and D) Diagrams illustrating that yields are calculated around individual species (A) and the entire community (D). The plots show the yields of acetate, H_2_, CO_2_, and formate for *S*. *fumaroxidans* (per propionate) (B and C); CH_4_ for *M*. *hungatei* (per CO_2_) (B and C); and acetate, H_2_, CO_2_, formate, and CH_4_ for the entire reactor (per propionate) (E and F), as a function of the dilution rate of the reactor (X-axis). Plots are shown in which H_2_ and CO_2_ and exchanged (B and E), and in which formate and H_2_ is exchanged (C and F).

Predicted yields around individual species were first evaluated (Fig 4A). At low dilution rates, *S*. *fumaroxidans* was predicted to convert propionate to acetate, H_2_, and CO_2_/formate and *M*. *hungatei* was predicted to convert CO_2_/formate to CH_4_ (Fig 4B and 4C, which show alternate solutions with CO_2_ or formate being exchanged). As the reactor dilution rate increased, the predicted species yields of H_2_ and CO_2_/formate (*S*. *fumaroxidans*) and CH_4_ (*M*. *hungatei*) decreased slightly (Fig 4B and 4C). In addition, species’ uptake rates of propionate and CO_2_/formate increased with dilution rate, as biochemical transformation of these substrates provides the energy needed for cellular growth and maintenance.

**Fig 5:**
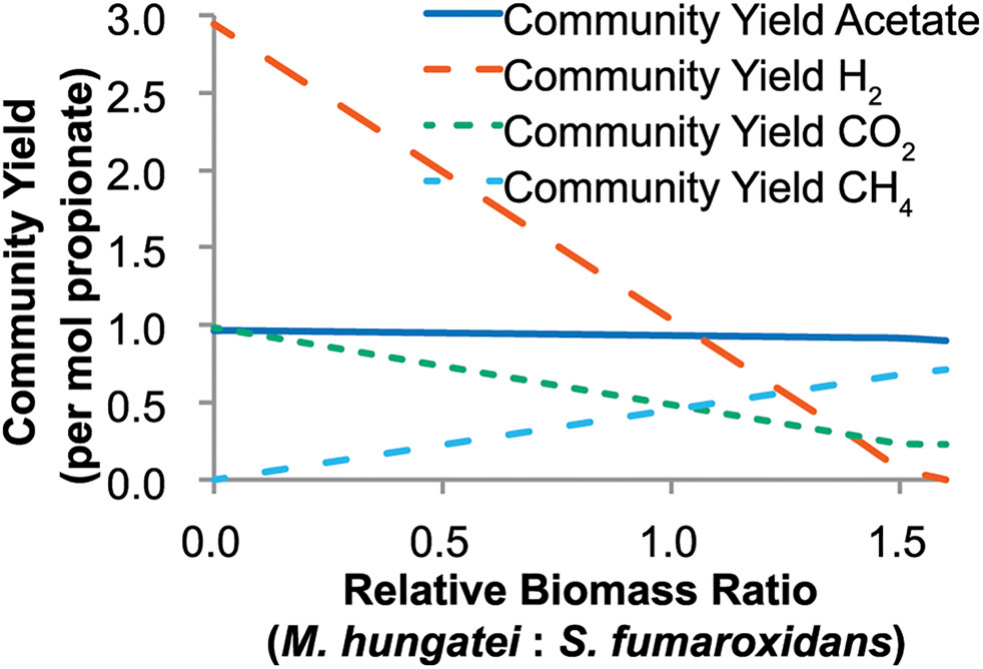
Predicted community by-product yields by the coculture at a dilution rate of 0.05 days^-1^. The plot shows the yields of acetate, H_2_, CO_2_, and CH_4_ for the entire reactor (per propionate) as a function of the relative biomass ratio of *M*. *hungatei* to *S*. *fumaroxidans* (X-axis). As in Fig 4, formate could be exchanged in the place of CO_2_ and H_2_.

Species’ uptake rates, secretion rates, and relative biomass ratio affects overall bioreactor yields, and these bioreactor yields were subsequently investigated (Fig 4D). At a 3:4 relative biomass ratio (*M*. *hungatei*: *S*. *fumaroxidans)*, *M*. *hungatei* did not fully utilize all of the H_2_ and CO_2_/formate produced by *S*. *fumaroxidans*, even at high dilution rates (Fig 4E and 4F). The net H_2_ production by the community indicates that *S*. *fumaroxidans* produces more H_2_ than *M*. *hungatei*’s needs and suggests the community can maintain higher *M*. *hungatei* to *S*. *fumaroxidans* ratios or that *S*. *fumaroxidans* could support faster growth of *M*. *hungatei*. At a dilution rate of 0.05 days^-1^, *S*. *fumaroxidans* produces H_2_ in excess of *M*. *hungatei*’s energy needs until the relative *M*. *hungatei* to *S*. *fumaroxidans* biomass ratio reaches approximately 1.6:1 (Fig 5). These simulations suggest that invariant external H_2_ concentration requires high ratios of *M*. *hungatei* to *S*. *fumaroxidans* (higher than has been proposed in the literature based on overall reaction stoichiometries), *M*. *hungatei* growing at faster rates than *S*. *fumaroxidans* (e.g., in batch culture), or a combination of the two.

Furthermore, studies have shown that in coculture, *S*. *fumaroxidans* passes electrons to *M*. *hungatei* via formate, as well as H_2_ [38,39,56]. Coculture simulations predicted that formate could be exchanged in lieu of CO_2_ (Fig 4B and 4C), without affecting the predicted bioreactor yields or species-weighted total flux (pTMFA objectives). When formate is exchanged the formate dehydrogenases of *S*. *fumaroxidans* and *M*. *hungatei* facilitate the interconversion of formate to CO_2_ and H_2_.

Finally, steady-state metabolite concentrations in the coculture were predicted using thermodynamic variability analysis [33,34] (S1 Text). Similar to a previous study of *E*. *coli* [34], the majority of steady-state metabolite concentrations were not constrained by thermodynamics (i.e., the concentration ranges were the global concentration bounds of 0.01mM and 20 mM). However, hypotheses for some extracellular (Table 2) and intracellular (S5 Table in S1 Dataset) metabolite concentrations could be made. The model predicts that propionate in the media must be greater than 0.36 mM for coculture growth to occur, and that acetate and CO_2_ concentrations must be less than 4mM and 0.65 mM, respectively. The model also predicts critical concentrations for H_2_ (0.0032 mM) and formate (0.0020 mM, when formate is being exchanged), which must be maintained in order for methanogenesis to occur. All of these predictions were insensitive to both the dilution rate and biomass ratio (*M*. *hungatei*: *S*. *fumaroxidans*).

**Table 2:** Model-predicted steady-state metabolite concentrations for select external metabolites in coculture.

Metabolite	Default Lower Bound	Default Upper Bound	Predicted Lower Bound	Predicted Upper Bound
Propionate	0.01 mM	20 mM	0.36 mM	20 mM
Acetate	0.01 mM	20 mM	0.01 mM	4.0 mM
Carbon Dioxide	0.01 mM	20 mM	0.27 mM	0.65 mM
H_2_	None*	20 mM	0.0032 mM	8.1 mM
Formate	None*	20 mM	0.0020 mM	4.0 mM
Methane	0.01 mM	20 mM	0.01 mM	20 mM

* No lower bounds were imposed on H_2_ or formate concentrations, which can fall below 0.01 mM, based on experimental measurements.

## Discussion

The *i*Mhu428 and *i*Sfu648 thermodynamic models were successfully used to verify proposed carbon and ATP production pathways in *M*. *hungatei* and *S*. *fumaroxidans;* however, some more efficient pathways (based on ATP or biomass yields) were found in some cases. The computational results highlighted topics that necessitate further discussion relating to (1) the stoichiometry of transport reactions, (2) the thermodynamics of H_2_ production, and (3) the carbon and electron shuttles between species in coculture.

### Ion Transport in the *i*Mhu428 Metabolic Model

The majority of the *i*Mhu428 model content comes from the *i*MB745 reconstruction of *M*. *acetivorans* and still needs to be verified. Changes to model content could affect the conclusions drawn about *M*. *hungatei* behavior. Despite containing a complete methanogenesis pathway, the *i*Mhu428 model was unable to identify the H^+^/Na^+^ transport stoichiometry of the energy-converting (Eha- or Ehb-type) hydrogenase (*EHA*, 1.12.7.2), which is thought to pump H^+^/Na^+^ while reducing ferredoxin [7]. The heterodisulfide reductase (*HDR*, 1.8.98.1) can also reduce ferredoxin, and the *i*Mhu428 model predicted *HDR* to be the only ferredoxin-reducing reaction required for methanogenesis. This observation is consistent with the observation that the expression of Eha/Ehb is considerably lower than that of *HDR* [57].

Additionally, different stoichiometries for other ion transport reactions important to methanogenesis remain thermodynamically possible. For example, the group contribution method predicted that tetrahydromethanopterin S-methyltransferase (*MTSPCMMT_CM5HBCMT*, E.C. 2.1.1.86) could drive transport of up to 4 Na^+^ ions under standard conditions, instead of the 2 Na^+^ ions used in the *i*Mhu428 reconstruction. Furthermore, some studies suggest the archaeal A_1_A_0_ ATP synthase is coupled to Na^+^ instead of H^+^ translocation [7,58]. When the *i*Mhu428 model was modified to reflect this coupling, the model predicted the Na^+^/H^+^ antiporter was no longer active, as Na^+^ ions from *MTSPCMMT_CM5HBCMT* were directly used for ATP synthesis. Thus, while the modeled methanogenesis pathway is consistent with available data, it is not the only possibility.

### H_2_ Production in the *i*Sfu648 Metabolic Model

Analysis of the *i*Sfu648 model revealed that H_2_ production is thermodynamically feasible in monoculture, implying there may be other biological reasons why H_2_ production is not normally observed under this condition, or that tighter estimates of thermodynamic parameters are needed. The *i*Sfu648 thermodynamic model also has some important limitations. In particular, the *i*Sfu648 model does not contain enough thermodynamic information to predict the directions of important electron transport reactions that involve ferredoxin, including the confurcating hydrogenase and formate dehydrogenase, the ferredoxin-oxidizing hydrogenase and formate dehydrogenase, and the RNF-type oxidoreductase (S3 Table in S1 Dataset). This is because the group contribution method is unable to estimate the standard transformed Gibbs free energy of formation (Δ_*f*_*G*^'0^) of ferredoxin, resulting in no Δ_*r*_*G*^'0^ estimates for these reactions. Fortunately, new quantum chemical approaches for estimating the thermodynamics of metabolism [59] may potentially provide additional Δ_*f*_*G*^'0^ estimates.

This work also raises important questions about the appropriate mathematical basis for representing thermodynamic constraints. Previous studies used the Δ*G*^'0^ of groups directly when modeling thermodynamics [34], and found that introducing uncertainty into a thermodynamic model of *E*. *coli* made the model computationally difficult to solve. In this work, using either the Δ*G*^'0^ of molecules or groups to model thermodynamics proved computationally difficult (results not shown). Instead, only using Δ_*r*_*G*^'0^ as the basis for thermodynamic calculations enabled uncertainties in free energy estimates to be handled without any computational difficulties. However, using Δ_*r*_*G*^'0^ as a basis for thermodynamic calculations leads to larger uncertainties in Δ_*r*_*G*^'0^ and greater network flexibility, as it does not account for thermodynamic interconnectivity between reactions with shared metabolites. As a result, the model-predicted feasible Δ_*r*_*G*^'0^ range through a linear combination of reactions considerably exceeds the group-contribution predicted Δ_*r*_*G*^'0^ range of the overall reaction. In the future, thermodynamic interconnectivity should be captured using the Δ*G*^'0^ of molecules or groups, and optimization techniques are needed to improve the runtime performance of the resulting thermodynamic models.

However, the use of Δ_*r*_*G*^'0^ as a basis for thermodynamic calculations is insufficient to explain why the *i*Sfu648 model could still produce H_2_ under monoculture growth conditions. As described in S1 Text, Δ_*f*_*G*^'0^ was used as a basis for thermodynamic calculations when attempting to find additional constraints which would prevent H_2_ production. The failure to find such constraints indicates either that thermodynamics does not explain the absence of H_2_ production in monoculture, or that current thermodynamic models cannot capture this phenomenon. If it is the latter, more accurate group contribution methods with smaller error estimates may eventually be able to explain the role of thermodynamics in syntrophic associations.

### Formate and H_2_ Transfer in Coculture

The thermodynamic coculture model of the syntrophic association between these species confirmed the role of formate and H_2_ in electron transfer in the community, and led us to hypothesize that total H_2_ consumption by the community indicates that *M*. *hungatei* cells are more abundant and/or faster growing than the *S*. *fumaroxidans* cells. The coculture model correctly predicted that both H_2_ and formate could shuttle electrons between members of this community. Formate may be preferred over H_2_ for electron transfer for thermodynamic reasons, as the Δ_*r*_*G*^'0^ of Eq 4 is more favorable (less positive) than that of Eq 3 in which formate is not exchanged. By exchanging formate in place of CO_2_ and H_2_, *S*. *fumaxoridans* could sustain propionate oxidation at higher extracellular concentrations of formate than of H_2_. Formate exchange could also be preferred due to differences in kinetics, diffusion, and/or volatility. These scenarios could stabilize the syntrophic association by enabling faster shuttling of electrons to *M*. *hungatei*.

This work highlights some important obstacles to successful modeling of microbial consortia. In order for computational models of microbial communities to make meaningful predictions, individual species models must be integrated into a community model in a biologically relevant manner. Such integration will require an understanding of the objectives and constraints governing the behavior of each community member, and this work demonstrates that identifying the proper constraints and objectives requires extensive experimental characterization of the community. For example, neither maximization of growth rate nor maximization of ATP yield were sufficient for the *i*Sfu648 model to predict the observed behavior of *S*. *fumaroxidans*. Additional constraints on reaction directions and flux ratios were required before *i*Sfu648 could be combined with *i*Mhu428 model to simulate the coculture. In addition, the *i*Sfu648 model relied on data from gene expression and ^13^C NMR experiments, suggesting that constraint-based approaches will complement traditional top down (‘omics’) approaches [60] by enabling a mechanistic understanding of microbial interactions [24].

## Methods

### Reconstruction of the *i*Mhu428 Metabolic Model

The *i*Mhu428 reconstruction of *M*. *hungatei* was built from the *i*MB745 reconstruction of *M*. *acetivorans* [41]. A preliminary draft reconstruction was built based on sequence homology (using the RAVEN Toolbox [42]), but the reconstruction contained less than 200 genes (S2 Dataset). Instead of performing extensive gapfilling, reactions from the *i*MB745 *M*. *acetivorans* reconstruction were copied into the *M*. *hungatei* reconstruction, with modifications to reflect key metabolic features of *M*. *hungatei* (see S1 Text). Results from the RAVEN Toolbox and the KEGG SSDB [43] were used to map genes in *M*. *acetivorans* to *M*. *hungatei* and identify those reactions which have genomic evidence (S2 Dataset). Finally, blocked reactions lacking genomic evidence were removed from the reconstruction.

The final *i*Mhu428 reconstruction contains 720 reactions, 428 genes (associated with 493 reactions), and 639 metabolites. Of the 428 genes, 351 were added based on sequence homology, and 77 were added manually. The reconstruction is available in S2 Dataset and S3 Dataset in Excel and SBML formats.

### Reconstruction of the *i*Sfu648 Metabolic Model

The *i*Sfu648 reconstruction of *S*. *fumaroxidans* was built from KEGG [43] (S6 Dataset and S7 Dataset) using the RAVEN Toolbox [42], which uses protein homology to identify the KEGG Orthology (KO) ID for each gene in a genome. The reactions and genes corresponding to that KO ID are then imported into the reconstruction. The resulting draft reconstruction was manually refined as described in S1 Text. The final *i*Sfu648 reconstruction contains 874 reactions, 648 genes (associated with 770 reactions), and 893 metabolites. The reconstruction is available in S4 Dataset and S5 Dataset, in Excel and SBML formats.

### Thermodynamics-Based Metabolic Flux Analysis (TMFA)

Flux-balance analysis (FBA) [27] is a constraint-based technique for predicting the state of a metabolic network consistent with physiochemical principles. FBA identifies a flux distribution which maximizes cellular growth (or some other objective function), subject to steady-state mass-balance and enzyme capacity constraints. Thermodynamics-Based Metabolic Flux Analysis (TMFA, [33,34]) extends FBA via the introduction of thermodynamic constraints, which require that the transformed Gibbs free energy of a reaction (Δ_*r*_*G*^'^) and its flux (*v*) have opposite signs. Estimates ($\Delta_{r}G_{est}^{'0}$) and uncertainties (${SE}_{\Delta_{r}G_{est}^{'0}}$) of Δ_*r*_*G*^'0^ for the reactions in the reconstructions were obtained using a group contribution method [61] via the von Bertalanffy 2.0 Toolbox [62]. TMFA was implemented as previously described [34], with additional details given in S1 Text. The mol files for metabolites in *i*Mhu428 and *i*Sfu648 are provided in S1 File and S2 File, respectively.

### Parsimonious TMFA (pTMFA)

pFBA [54] is a constraint-based approach which maximizes cellular growth while also minimizing total flux through the network (a proxy for minimizing the total mass of enzymes required to sustain optimal growth through the network). pTMFA uses the same assumptions as pFBA while implementing the thermodynamic constraints of TMFA. pTMFA was implemented as a two-stage optimization process. In the first stage, growth rate is maximized via TMFA. In the second stage, the growth rate is fixed and the total flux through the network is minimized, subject to the same constraints as TMFA. Additional details on implementation can be found in S1 Text.

### Coculture Model

For the coculture simulations, a community model of growth in a continuous stirred-tank reactor was developed that accounts for the biomass concentrations of each species. The model minimizes the species-weighted total flux through the metabolic networks subject to TMFA constraints for each species and mass balances around the entire reactor. Details on the specific implementation used in this work can be found in S1 Text. To avoid solving a mixed-integer non-linear program (MINLP), the dilution rate and biomass concentrations for each species were fixed, resulting in a MIP. To explore the community behavior under a variety of operating conditions, the reactor dilution rate was systematically changed, while allowing unlimited propionate uptake by the reactor.

### Simulations

The uptake fluxes for carbon and other nutrients used in the simulations are given in S1 Table in S1 Dataset. All simulations were performed using CPLEX 12 (IBM, Armonk, NY) accessed via the General Algebraic Modeling System, Version 23.9.5 (GAMS, GAMS Development Corporation, Washington, DC). Estimates ($\Delta_{r}G_{est}^{'0}$) and uncertainties (${SE}_{\Delta_{r}G_{est}^{'0}}$) of thermodynamic parameters were obtained using version 2.0 of von Bertalanffy and Matlab R2012b (The MathWorks, Inc., Natick, MA).

## Supporting Information

S1 Dataset Supporting tables. This file contains Supporting Tables S1 to S7 as described in the text.
(XLS)

S2 Dataset Excel version of the *i*Mhu428 model. (XLS)

S3 Dataset SBML version of the *i*Mhu428 model. (XML)

S4 Dataset Excel version of the *i*Sfu648 model. (XLS)

S5 Dataset SBML version of the *i*Sfu648 model. (XML)

S6 Dataset Excel version of the balanced KEGG database. (XLS)

S7 Dataset SBML version of the balanced KEGG database. (XML)

S1 File Molfile structure files for all metabolites in the iMhu428 GEM. (ZIP)

S2 File Molfile structure files for all metabolites in the iSfu648 GEM. (ZIP)

S1 Text Supporting text. This file contains Supporting Methods, Results, and Figs to complement the main text.
(PDF)

## References

[1] Schink B (2002) Synergistic interactions in the microbial world. Antonie Van Leeuwenhoek 81: 257–261. 12448724 10.1023/a:1020579004534

[2] McInerney MJ, Struchtemeyer CG, Sieber JR, Mouttaki H, Stams AJM, et al (2008) Physiology, ecology, phylogeny, and genomics of microorganisms capable of syntrophic metabolism. Ann N Y Acad Sci 1125: 58–72. 10.1196/annals.1419.005 18378587

[3] Stams AJM, Plugge CM (2009) Electron transfer in syntrophic communities of anaerobic bacteria and archaea. Nat Rev Microbiol 7: 568–577. 10.1038/nrmicro2166 19609258

[4] McInerney MJ, Sieber JR, Gunsalus RP (2009) Syntrophy in anaerobic global carbon cycles. Curr Opin Biotechnol 20: 623–632. 10.1016/j.copbio.2009.10.001 19897353 PMC2790021

[5] Schink B (1997) Energetics of syntrophic cooperation in methanogenic degradation. Microbiol Mol Biol Rev 61: 262–280. 9184013 10.1128/mmbr.61.2.262-280.1997 PMC232610

[6] Stams AJM, de Bok FAM, Plugge CM, van Eekert MHA, Dolfing J, et al (2006) Exocellular electron transfer in anaerobic microbial communities. Environ Microbiol 8: 371–382. 16478444 10.1111/j.1462-2920.2006.00989.x

[7] Thauer RK, Kaster A-K, Seedorf H, Buckel W, Hedderich R (2008) Methanogenic archaea: ecologically relevant differences in energy conservation. Nat Rev Microbiol 6: 579–591. 10.1038/nrmicro1931 18587410

[8] Müller N, Worm P, Schink B, Stams AJM, Plugge CM (2010) Syntrophic butyrate and propionate oxidation processes: from genomes to reaction mechanisms. Environ Microbiol Rep 2: 489–499. 10.1111/j.1758-2229.2010.00147.x 23766220

[9] Sieber JR, McInerney MJ, Gunsalus RP (2012) Genomic insights into syntrophy: the paradigm for anaerobic metabolic cooperation. Annu Rev Microbiol 66: 429–452. 10.1146/annurev-micro-090110-102844 22803797

[10] Oberhardt MA, Palsson BØ, Papin JA (2009) Applications of genome-scale metabolic reconstructions. Mol Syst Biol 5: 320 10.1038/msb.2009.77 19888215 PMC2795471

[11] Lewis NE, Nagarajan H, Palsson BØ (2012) Constraining the metabolic genotype-phenotype relationship using a phylogeny of in silico methods. Nat Rev Microbiol 10: 291–305. 10.1038/nrmicro2737 22367118 PMC3536058

[12] Zomorrodi AR, Suthers PF, Ranganathan S, Maranas CD (2012) Mathematical optimization applications in metabolic networks. Metab Eng 14: 672–686. 10.1016/j.ymben.2012.09.005 23026121

[13] Mahadevan R, Palsson BØ, Lovley DR (2011) In situ to in silico and back: elucidating the physiology and ecology of Geobacter spp. using genome-scale modelling. Nat Rev Microbiol 9: 39–50. 10.1038/nrmicro2456 21132020

[14] Osterlund T, Nookaew I, Nielsen J (2012) Fifteen years of large scale metabolic modeling of yeast: Developments and impacts. Biotechnol Adv 30: 979–988. 10.1016/j.biotechadv.2011.07.021 21846501

[15] McCloskey D, Palsson BØ, Feist AM (2013) Basic and applied uses of genome-scale metabolic network reconstructions of Escherichia coli. Mol Syst Biol 9: 661 10.1038/msb.2013.18 23632383 PMC3658273

[16] Stolyar SM, Van Dien SJ, Hillesland KL, Pinel N, Lie TJ, et al (2007) Metabolic modeling of a mutualistic microbial community. Mol Syst Biol 3: 92 17353934 10.1038/msb4100131 PMC1847946

[17] Wintermute EH, Silver PA (2010) Emergent cooperation in microbial metabolism. Mol Syst Biol 6: 407 10.1038/msb.2010.66 20823845 PMC2964121

[18] Freilich S, Zarecki R, Eilam O, Segal ES, Henry CS, et al (2011) Competitive and cooperative metabolic interactions in bacterial communities. Nat Commun 2: 589 10.1038/ncomms1597 22158444

[19] Klitgord N, Segrè D (2010) Environments that induce synthetic microbial ecosystems. PLoS Comput Biol 6: e1001002 10.1371/journal.pcbi.1001002 21124952 PMC2987903

[20] Bordbar A, Lewis NE, Schellenberger J, Palsson BØ, Jamshidi N (2010) Insight into human alveolar macrophage and M. tuberculosis interactions via metabolic reconstructions. Mol Syst Biol 6: 422 10.1038/msb.2010.68 20959820 PMC2990636

[21] Lewis NE, Schramm G, Bordbar A, Schellenberger J, Andersen MP, et al (2010) Large-scale in silico modeling of metabolic interactions between cell types in the human brain. Nat Biotechnol 28: 1279–1285. 10.1038/nbt.1711 21102456 PMC3140076

[22] Heinken A, Sahoo S, Fleming RMT, Thiele I (2013) Systems-level characterization of a host-microbe metabolic symbiosis in the mammalian gut. Gut Microbes 4: 28–40. 10.4161/gmic.22370 23022739 PMC3555882

[23] Shoaie S, Karlsson FH, Mardinoglu A, Nookaew I, Bordel S, et al (2013) Understanding the interactions between bacteria in the human gut through metabolic modeling. Sci Rep 3: 2532 10.1038/srep02532 23982459 PMC3755282

[24] Nagarajan H, Embree M, Rotaru A-E, Shrestha PM, Feist AM, et al (2013) Characterization and modelling of interspecies electron transfer mechanisms and microbial community dynamics of a syntrophic association. Nat Commun 4: 2809 10.1038/ncomms3809 24264237

[25] Zomorrodi AR, Maranas CD (2012) OptCom: A Multi-Level Optimization Framework for the Metabolic Modeling and Analysis of Microbial Communities. PLoS Comput Biol 8: e1002363 10.1371/journal.pcbi.1002363 22319433 PMC3271020

[26] Zomorrodi AR, Islam MM, Maranas CD (2014) d-OptCom: Dynamic multi-level and multi-objective metabolic modeling of microbial communities. ACS Synth Biol 3: 247–257. 10.1021/sb4001307 24742179

[27] Orth JD, Thiele I, Palsson BØ (2010) What is flux balance analysis? Nat Biotechnol 28: 245–248. 10.1038/nbt.1614 20212490 PMC3108565

[28] Khandelwal RA, Olivier BG, Röling WFM, Teusink B, Bruggeman FJ (2013) Community flux balance analysis for microbial consortia at balanced growth. PLoS One 8: e64567 10.1371/journal.pone.0064567 23741341 PMC3669319

[29] Beard DA, Liang S, Qian H (2002) Energy balance for analysis of complex metabolic networks. Biophys J 83: 79–86. 12080101 10.1016/S0006-3495(02)75150-3 PMC1302128

[30] Qian H, Beard DA, Liang S (2003) Stoichiometric network theory for nonequilibrium biochemical systems. Eur J Biochem 270: 415–421. 12542691 10.1046/j.1432-1033.2003.03357.x

[31] Schellenberger J, Lewis NE, Palsson BØ (2011) Elimination of thermodynamically infeasible loops in steady-state metabolic models. Biophys J 100: 544–553. 10.1016/j.bpj.2010.12.3707 21281568 PMC3030201

[32] Kümmel A, Panke S, Heinemann M, Kummel A (2006) Putative regulatory sites unraveled by network-embedded thermodynamic analysis of metabolome data. Mol Syst Biol 2: 2006.0034. 10.1038/msb4100074 PMC1681506 16788595

[33] Henry CS, Broadbelt LJ, Hatzimanikatis V (2007) Thermodynamics-based metabolic flux analysis. Biophys J 92: 1792–1805. 17172310 10.1529/biophysj.106.093138 PMC1796839

[34] Hamilton JJ, Dwivedi V, Reed JL (2013) Quantitative assessment of thermodynamic constraints on the solution space of genome-scale metabolic models. Biophys J 105: 512–522. 10.1016/j.bpj.2013.06.011 23870272 PMC3714879

[35] Dolfing J (1987) Microbiological aspects of granular methanogenic sludge Wageningen Agricultural University.

[36] Stams AJM, Van Dijk JB, Dijkema C, Plugge CM (1993) Growth of syntrophic propionate-oxidizing bacteria with fumarate in the absence of methanogenic bacteria. Appl Environ Microbiol 59: 1114–1119. 16348912 10.1128/aem.59.4.1114-1119.1993 PMC202247

[37] Harmsen HJM, Van Kuijk BLM, Plugge CM, Akkermans ADL, De Vos WM, et al (1998) Syntrophobacter fumaroxidans sp. nov., a syntrophic propionate-degrading sulfate-reducing bacterium. Int J Syst Bacteriol 48: 1383–1387. 9828440 10.1099/00207713-48-4-1383

[38] Dong X, Stams AJM (1995) Evidence for H2 and formate formation during syntrophic butyrate and propionate degradation. Anaerobe 1: 35–39. 16887505 10.1016/s1075-9964(95)80405-6

[39] Stams AJM, Dong X (1995) Role of formate and hydrogen in the degradation of propionate and butyrate by defined suspended cocultures of acetogenic and methanogenic bacteria. Antonie Van Leeuwenhoek 68: 281–284. 8821782 10.1007/BF00874137

[40] Dong X, Plugge CM, Stams AJM (1994) Anaerobic degradation of propionate by a mesophilic acetogenic bacterium in coculture and triculture with different methanogens. Appl Environ Microbiol 60: 2834–2838. 16349350 10.1128/aem.60.8.2834-2838.1994 PMC201730

[41] Benedict MN, Gonnerman MC, Metcalf WW, Price ND (2012) Genome-scale metabolic reconstruction and hypothesis testing in the methanogenic archaeon Methanosarcina acetivorans C2A. J Bacteriol 194: 855–865. 10.1128/JB.06040-11 22139506 PMC3272958

[42] Agren R, Liu L, Shoaie S, Vongsangnak W, Nookaew I, et al (2013) The RAVEN Toolbox and Its Use for Generating a Genome-scale Metabolic Model for Penicillium chrysogenum. PLoS Comput Biol 9: e1002980 10.1371/journal.pcbi.1002980 23555215 PMC3605104

[43] Kanehisa M, Goto S (2000) KEGG: Kyoto Encyclopedia of Genes and Genomes. Nucleic Acids Res 28: 27–30. 10592173 10.1093/nar/28.1.27 PMC102409

[44] Schwörer B, Thauer RK (1991) Activities of formylmethanofuran dehydrogenase, methylenetetrahydromethanopterin dehydrogenase, methylenetetrahydromethanopterin reductase, and heterodisulfide reductase in methanogenic bacteria. Arch Microbiol 155: 459–465.

[45] Thauer RK (1998) Biochemistry of methanogenesis: a tribute to Marjory Stephenson. Microbiology 144: 2377–2406. 9782487 10.1099/00221287-144-9-2377

[46] Ferry JG (1999) Enzymology of one-carbon metabolism in methanogenic pathways. FEMS Microbiol Rev 23: 13–38. 10077852 10.1111/j.1574-6976.1999.tb00390.x

[47] Liu Y, Whitman WB (2008) Metabolic, phylogenetic, and ecological diversity of the methanogenic archaea. Ann N Y Acad Sci 1125: 171–189. 10.1196/annals.1419.019 18378594

[48] Plugge CM, Henstra AM, Worm P, Swarts DC, Paulitsch-Fuchs AH, et al (2012) Complete genome sequence of Syntrophobacter fumaroxidans strain (MPOB(T)). Stand Genomic Sci 7: 91–106. 10.4056/sigs.2996379 23450070 PMC3570798

[49] De Bok FAM, Roze EHA, Stams AJM (2002) Hydrogenases and formate dehydrogenases of Syntrophobacter fumaroxidans. Antonie Van Leeuwenhoek 81: 283–291. 12448727 10.1023/a:1020539323190

[50] De Bok FAM, Hagedoorn P-L, Silva PJ, Hagen WR, Schiltz E, et al (2003) Two W-containing formate dehydrogenases (CO2-reductases) involved in syntrophic propionate oxidation by Syntrophobacter fumaroxidans. Eur J Biochem 270: 2934–2942. 10.1046/j.1432-1033.2003.03619.x 12755703

[51] Worm P, Stams AJM, Cheng X, Plugge CM (2011) Growth- and substrate-dependent transcription of formate dehydrogenase and hydrogenase coding genes in Syntrophobacter fumaroxidans and Methanospirillum hungatei. Microbiology 157: 280–289. 10.1099/mic.0.043927-0 20884694

[52] Plugge CM, Dijkema C, Stams AJM (1993) Acetyl-CoA cleavage pathway in a syntrophic propionate oxidizing bacterium growing on fumarate in the absence of methanogens. FEMS Microbiol Lett 110: 71–76.

[53] Scholten JCM, Conrad R (2000) Energetics of syntrophic propionate oxidation in defined batch and chemostat cocultures. Appl Environ Microbiol 66: 2934–2942. 10877789 10.1128/aem.66.7.2934-2942.2000 PMC92094

[54] Lewis NE, Hixson KK, Conrad TM, Lerman JA, Charusanti P, et al (2010) Omic data from evolved E. coli are consistent with computed optimal growth from genome-scale models. Mol Syst Biol 6: 390 10.1038/msb.2010.47 20664636 PMC2925526

[55] Ekiel I, Smith ICP, Sprott GD (1983) Biosynthetic pathways in Methanospirillum hungatei as determined by 13C nuclear magnetic resonance. J Bacteriol 156: 316–326. 6619097 10.1128/jb.156.1.316-326.1983 PMC215085

[56] De Bok FAM, Luijten MLGC, Stams AJM (2002) Biochemical Evidence for Formate Transfer in Syntrophic Propionate-Oxidizing Cocultures of Syntrophobacter fumaroxidans and Methanospirillum hungatei. Appl Environ Microbiol 68: 4247–4252. 12200272 10.1128/AEM.68.9.4247-4252.2002 PMC124120

[57] Tersteegen A, Hedderich R (1999) Methanobacterium thermoautotrophicum encodes two multisubunit membrane-bound [NiFe] hydrogenases. Transcription of the operons and sequence analysis of the deduced proteins. Eur J Biochem 264: 930–943. 10491142 10.1046/j.1432-1327.1999.00692.x

[58] Pisa KY, Huber H, Thomm M, Müller V (2007) A sodium ion-dependent A1AO ATP synthase from the hyperthermophilic archaeon Pyrococcus furiosus. FEBS J 274: 3928–3938. 17614964 10.1111/j.1742-4658.2007.05925.x

[59] Jinich A, Rappoport D, Dunn I, Sanchez-Lengeling B, Olivares-Amaya R, et al (2014) Quantum Chemical Approach to Estimating the Thermodynamics of Metabolic Reactions. Sci Rep 4: 7022 10.1038/srep07022 25387603 PMC5381496

[60] Zarraonaindia I, Smith DP, Gilbert JA (2013) Beyond the genome: community-level analysis of the microbial world. Biol Philos 28: 261–282. 23482824 10.1007/s10539-012-9357-8 PMC3585761

[61] Noor E, Haraldsdóttir HS, Milo R, Fleming RMT (2013) Consistent Estimation of Gibbs Energy Using Component Contributions. PLoS Comput Biol 9: e1003098 10.1371/journal.pcbi.1003098 23874165 PMC3708888

[62] Schellenberger J, Que R, Fleming RMT, Thiele I, Orth JD, et al (2011) Quantitative prediction of cellular metabolism with constraint-based models: the COBRA Toolbox v2.0. Nat Protoc 6: 1290–1307. 10.1038/nprot.2011.308 21886097 PMC3319681

