# Supplementary material for: Thermodynamics and H2 Transfer in a Methanogenic, Syntrophic Community
Source: PLoS Comput Biol. 2015 Jul 6;11(7):e1004364. doi: 10.1371/journal.pcbi.1004364 (PMC4509577; doi:10.1371/journal.pcbi.1004364)
Supplement: S1 Text — This file contains Supporting Methods, Results, and Figs to complement the main text. (PDF) [file pcbi.1004364.s010.pdf]

Supporting Materials for “Thermodynamics and H_2 Transfer in a Methanogenic, Syntrophic Community”

Joshua J. Hamilton[1], Montserrat Calixto Contreras[1], and Jennifer L. Reed[1,§]

[1]Department of Chemical and Biological Engineering, University of Wisconsin-Madison, Madison, WI, USA
[§]Corresponding author

Email addresses:
JJH: jjhamilton2@wisc.edu
MCC: calixto@wisc.edu
JLR: reed@engr.wisc.edu

Table of Contents

1 Supporting Results 1
1.1 Testing and Parameterizing the *i*Mhu428 Metabolic Model 1
1.2 Testing and Parameterizing the *i*Sfu648 Metabolic Model 2
1.2.1 Elimination of Thermodynamically Infeasible Closed Cycles 2
1.2.2 ATP Production Mechanisms in *S. fumaroxidans* 2
1.2.3 Validation of ATP Production Mechanisms 3
1.2.4 Thermodynamics of H_2 Production 4
2 Supporting Discussion 6
2.1 Testing and Parameterizing of the *i*Sfu648 Metabolic Model 6
3 Supporting Methods 7
3.1 Reconstruction and Parameterization of the *i*Mhu428 Metabolic Model 7
3.2 Reconstruction and Parameterization of the *i*Sfu648 Metabolic Model 8
3.2.1 Curation of Draft *i*Sfu648 Model 8
3.2.2 Manual Curation of Energy Conservation Mechanisms 9
3.2.3 Manual Curation of Carbon Transfer Mechanisms 11
3.2.4 Experimental Justification for Theoretical Flux Distributions 11
3.2.5 Regulatory Constraints 12
3.2.6 Development and Gapfilling of Biomass Equation 13
3.2.7 Gapfilling of Blocked Reactions 14
3.2.8 Conversion to a Thermodynamic Model 14
3.2.9 *i*Sfu648 Model Parameterization 14
3.3 Metabolite Structures and Molecular Formulas 15
3.4 Thermodynamics-Based Metabolic Flux Analysis (TMFA) 15
3.5 TMFA and Thermodynamics of H_2 Production 17
3.6 Thermodynamic Lumping 18
3.7 Linear Programming Approximation of TMFA (TMFA-LP) 19
3.8 Parsimonious TMFA (pTMFA) 19
3.9 Community Formulation 20
3.10 Community Thermodynamic Variability Analysis 22
3.11 Minimal Probabilistic Sets (MPS) 22

3.11.1 MPS for Validating *i*Mhu428: ATP Gain 24
3.11.2 MPS for Validating *i*Mhu428: Biomass Growth...25
3.11.3 MPS for Validating *i*Sfu648: Closed-Network ATP Cycles 25
3.11.4 MPS for Validating *i*Sfu648: ATP Gain 26
3.12 Simulation Conditions 26
4 Supporting Figures 27
4.1 Fig S1 27
4.2 Fig S2 28
5 Supporting References 29

1 Supporting Results

1.1 Testing and Parameterizing the *i*Mhu428 Metabolic Model

Methanogens can be classified into two groups based on the presence or absence of cytochromes in their energy transfer mechanisms [7]. To date, GEMs have been constructed for two methanogens, *Methanosarcina barkeri* [63,64] and *Methanosarcina acetivorans* [41,65], both of which use cytochromes. To the best of our knowledge, there are no published GEMs for methanogens lacking cytochromes, such as *M. hungatei*.

After the *i*Mhu428 reconstruction was converted to a thermodynamic model, TMFA predicted a no-growth phenotype, due to the inability of the *i*Mhu428 model to oxidize H_2S to SO_3^{2-} via sulfite reductase, a necessary reaction for biomass production. The estimated $\Delta_r G'$ for this reaction was between 55.6 kJ/mol and 284 kJ/mol, indicating that the reaction could not proceed in the direction required for growth. However, replacing the coenzyme F_{420} with a generic ferredoxin enabled sulfite reductase to proceed in the required direction. Because sulfur metabolism in methanogens remains poorly understood [66], this difference in sulfur metabolism between *M. acetivorans* and *M. hungatei* seems plausible.

The *i*Mhu428 model predicted that *M. hungatei* is able to generate 0.5 mol ATP per mol of CO_2 converted to CH_4. However, the *i*Mhu428 model predicted other, higher-yielding, ATP-generating mechanisms outside the methanogenesis pathway, despite experimental evidence suggesting methanogenesis is the sole source of ATP. The enumeration of all such ATP-generating cycles [67] proved computationally intractable, so a previously proposed probability-based approach [68] was used to qualitatively constrain reaction directions in the *i*Mhu428 model. This approach calculates the probability that a reaction's standard transformed Gibbs free energy of reaction ($\Delta_r G'^0$) is negative. If the probability is greater (less) than 70% (30%), the reaction is constrained to the forward (reverse) direction. Constraining all 497 such reactions eliminated these higher ATP yielding mechanisms, but also resulted in a no-growth phenotype.

To overcome this problem, a new optimization approach was developed (see Minimal Probabilistic Sets, or MPS). MPS minimizes the number of qualitative reaction direction constraints needed to reduce the maximum ATP gain to 0.5 mol ATP/mol CO_2, while still allowing for growth. A set of 41 reactions had to be constrained to a single direction (listed in Table S2 in S1 Dataset of the Supporting Material). Many of the reactions identified by MPS may be thermodynamically unidirectional *in vivo* due to intracellular metabolite concentrations related to the cellular energy charge [69], but these constraints are not captured in the *i*Mhu428 model. These additional reaction direction constraints were used in all subsequent analyses with the *i*Mhu428 model.

It has also been observed that biomass and energy generation are independent processes in *M. hungatei*, with CO_2 being the sole source of ATP (using methanogenesis), and acetate being the sole source of carbon for biomass [55]. In contrast, the *i*Mhu428 model predicts that *M. hungatei* can produce some biomass using CO_2 alone, and MPS could not identify any qualitative reaction direction

constraints which would prevent CO_2 from being used to generate biomass while still allowing biomass production from acetate (see Supporting Methods for additional details). Nonetheless, during growth on CO_2 and acetate, over 90% of the CO_2 consumed is used for methanogenesis.

1.2 Testing and Parameterizing the *i*Sfu648 Metabolic Model

1.2.1 Elimination of Thermodynamically Infeasible Closed Cycles

A thermodynamically consistent metabolic network should contain no thermodynamically infeasible closed cycles [29]. Such cycles may become problematic if they enable undesirable behaviors, such as free ATP production. The thermodynamic model for *i*Sfu648 allowed ATP producing cycles when no nutrients were provided. As with the *i*Mhu428 reconstruction, MPS was used to minimize the number of qualitative reaction direction constraints needed to eliminate these cycles, while maintaining biomass growth. The resulting qualitative reaction direction constraints are given in Table S2 in S1 Dataset of the Supporting Material.

1.2.2 ATP Production Mechanisms in *S. fumaroxidans*

Experimental studies have elucidated five growth modes for *S. fumaroxidans*: four in monoculture and one in coculture with *M. hungatei* [36,48,52]. In monoculture, *S. fumaroxidans* can convert fumarate to succinate and CO_2. The bacterium can also grow on fumarate with formate or H_2 as an electron donor, producing succinate as the only by-product. *S. fumaroxidans* can also oxidize propionate to succinate using fumarate as an electron acceptor. A final monoculture growth mode has been observed in which *S. fumaroxidans* couples propionate oxidation to sulfate reduction [70]. However, the sulfate reduction pathway in *S. fumaroxidans* has not been fully characterized [48]. In coculture with *M. hungatei*, *S. fumaroxidans* grows on propionate and produces acetate, H_2 and CO_2/formate; however, *S. fumaroxidans* can not grow on propionate alone in monoculture. This work examines the most commonly studied growth modes: monoculture growth on fumarate (Fig 2A), monoculture growth on fumarate plus propionate (Fig 2B), and coculture growth on propionate (Fig 2C).

During monoculture growth on fumarate alone (Fig 2A), one mole of fumarate gets fully oxidized to CO_2, while six moles of fumarate get reduced to succinate [48,52]:

$$7 \text{ fumarate} \rightarrow 6 \text{ succinate} + 4\, CO_2 \qquad \text{(S1)}$$

The oxidation of one fumarate to CO_2 generates one ATP and five reducing equivalents (three NADH and two pairs of reduced ferredoxin) [48,52], while the reduction of additional fumarate to succinate by fumarate reductase (*FRD*) produces menaquinone [48]. The Rnf complex (*RNF*) reoxidizes NADH via ferredoxin reduction, while the ferredoxin-oxidizing hydrogenase (*frH$_2$ase*) transfers electrons from ferredoxin to protons, generating H_2 and oxidized ferredoxin. The cytosolic hydrogenase (*cytH$_2$ase*) then transfers electrons from H_2 to menaquinone, generating menaquinol. An additional pair of electrons (in the form of H_2) is provided to *cytH$_2$ase* via formate hydrogen lyase (*FHL*), the final step in fumarate oxidation to CO_2. Reduction of fumarate to succinate recycles menaquinol back into menaquinone and generates the proton motive force (PMF) responsible for driving the *RNF* reaction and producing ATP.

During monoculture growth on fumarate plus propionate (Fig 2B), one mole of propionate gets oxidized to succinate, while one mole of fumarate gets oxidized to acetate and CO_2. Two additional moles of fumarate get reduced to succinate [36,48]:

$$\text{propionate} + 3\ \text{fumarate} \rightarrow \text{acetate} + CO_2 + 3\ \text{succinate} \qquad \text{(S2)}$$

The oxidation of fumarate to acetate and CO_2 produces one NADH and one pair of reduced ferredoxin. The confurcating hydrogenase (*cH₂ase*) couples NADH and ferredoxin oxidation with H_2 production. The cytosolic hydrogenase (*cytH₂ase*) then transfers electrons from H_2 to menaquinone, generating menaquinol and driving fumarate reductase (*FRD*). Oxidation of propionate to succinate produces one ATP, while *FRD* generates the PMF necessary for additional ATP production.

During coculture growth on propionate (Fig 2C), propionate gets oxidized to acetate and CO_2 via the methylmalonyl-CoA pathway [48,52].

$$\text{propionate} \rightarrow \text{acetate} + CO_2 + 3\ H_2 \qquad \text{(S3)}$$

A single ATP is generated during the oxidation of propionate to succinate. This ATP is hydrolyzed to generate the PMF necessary for the endergonic oxidation of succinate to fumarate (*SDH*). This oxidation generates menaquinol; the cytosolic hydrogenase (*cytH₂ase*) then transfers electrons from menaquinol to two protons, generating H_2. The further oxidation of fumarate to acetate and CO_2 produces one NADH and one pair of reduced ferredoxin. The confurcating hydrogenase (*cH₂ase*) couples NADH and ferredoxin re-oxidation with H_2 production. Unlike in the monoculture growth modes, the H_2 is not consumed by the cytosolic hydrogenase and must diffuse outside the cell. It has been proposed that the net production of H_2 by *S. fumaroxidans* is only thermodynamically favorable at the low H_2 concentrations maintained by methanogens, thereby explaining why *S. fumaroxidans* only produces H_2 during coculture growth.

S. fumaroxidans exhibits considerable flexibility in its ATP production mechanisms during syntrophic growth ([8,9,48,51]), and can produce formate instead of CO_2 (Fig 3) yielding an overall transformation of:

$$\text{propionate} \rightarrow \text{acetate} + \text{formate} + 2\ H_2 \qquad \text{(S4)}$$

In one mechanism (Fig 3A), activity of the cytosolic formate hydrogenase (*cytFDH*) substitutes for the activity of the *cytH₂ase.* CO_2 produced by the oxidation of propionate serves as a substrate for *cytFDH*, which transfers electrons from menaquinol to CO_2, generating formate. In a second mechanism (Fig 3B), the confurcating formate dehydrogenase (*cFDH*) substitutes for the *cH₂ase*. Here, the *cFDH* couples NADH and ferredoxin re-oxidation with the conversion of CO_2 (from propionate oxidation) to formate.

1.2.3 Validation of ATP Production Mechanisms

Experimental evidence suggests that the carbon and electron transport mechanisms described in Table 2 and Table S3 (in S1 Dataset in the Supporting Material) provide the sole source for ATP production in *S. fumaroxidans*, either by substrate-level phosphorylation or through establishment of a proton gradient used by ATP synthase [5]. However, the *i*Sfu648 model includes additional pathways for ATP synthesis with higher ATP yields. To identify these pathways, the *i*Sfu648 model was first used to identify the highest amount of ATP that could be produced using the experimentally known mechanisms. The *i*Sfu648 model was then analyzed to find and eliminate other higher ATP yielding pathways.

As with the *i*Mhu428 reconstruction, mechanisms with higher ATP yields were identified, and MPS was used to eliminate them. For each growth condition, MPS identified the smallest number of qualitative reaction direction constraints needed to maintain biomass growth while ensuring the appropriate maximal ATP gain. The resulting qualitative reaction direction constraints are given in Table S2 in S1 Dataset of the Supporting Material. Many of the reactions identified by MPS may be thermodynamically unidirectional *in vivo* due to intracellular metabolite concentrations related to the cellular energy charge [69], but these constraints are not captured in the *i*Sfu648 model. These additional reaction direction constraints were used in all subsequent analyses with the *i*Sfu648 model.

1.2.4 Thermodynamics of H_2 Production

Experimental studies of *S. fumaroxidans* have shown that H_2 is not produced during growth in monoculture [36,52,53]. However, our simulations reveal that H_2 production remains thermodynamically feasible under monoculture conditions. For example, during monoculture growth on fumarate, the *i*Sfu648 model predicts that H_2 can be produced via the following mechanism:

$$\text{fumarate} \rightarrow 4\ CO_2 + 6\ H_2 \qquad \text{(S5)}$$

In this scenario, H_2 molecules produced by the ferredoxin-oxidizing hydrogenase are exported outside the cell, instead of serving as substrates for the cytosolic hydrogenase. As a result, no proton motive force is generated by fumarate reductase, and the net ATP yield is zero. This suggests that thermodynamic considerations alone cannot explain the absence of H_2 production during monoculture growth, but that the observed flux distribution may instead be driven by demands for energy generation.

To determine if thermodynamics could play a role in explaining the absence of H_2 production in monoculture conditions, we sought to identify a constraint on extracellular metabolite concentrations that would eliminate H_2 production [34] (e.g., if extracellular CO_2 and H_2 concentrations are too high, H_2 production would become thermodynamically unfavorable). Such a constraint was identified, but the iSfu648 model predicted a shift from CO_2 and H_2 to formate production:

$$\text{fumarate} \rightarrow 3\ CO_2 + 5\ H_2 + \text{for} \qquad \text{(S6)}$$

Once again, a constraint on extracellular CO_2, H_2, and formate concentration was identified to render this extracellular flux distribution thermodynamically unfeasible, and the *i*Sfu648 model predicted fumarate reduction to succinate:

$$\text{fumarate} \rightarrow 2\ CO_2 + 0.5\ \text{succinate} + 2.5\ H_2 \qquad \text{(S7)}$$

However, no constraint could be found which would fully eliminate H_2 production.

Similar results were obtained during monoculture growth during simultaneous utilization of fumarate and propionate. The *i*Sfu648 model predicted that H_2 could be produced via the following mechanism:

$$\text{propionate} + \text{fumarate} \rightarrow CO_2 + \text{acetate} + \text{succinate} + 2\ H_2 \qquad \text{(S8)}$$

In this scenario, propionate is oxidized to succinate, while fumarate is oxidized to CO_2 and acetate, producing NADH and reduced ferredoxin. The confucrating hydrogenase reoxidizes ferredoxin and NADH, producing H_2. The H_2 is exported outside the cell, instead of participating in fumarate reduction as a substrate for the cytosolic hydrogenase. The net ATP yield is one mole ATP per mole propionate, less than the maximum ATP yield of 2 1/3 ATP per mole propionate, which occurs when fumarate is

reduced. Again, this suggests that the observed flux distribution may be driven by demands for energy generation instead of thermodynamics.

Furthermore, no constraint on metabolite concentrations could be identified which would make this H_2-production mechanism thermodynamically infeasible. Thus, if the absence of H_2 production under monoculture conditions is governed by thermodynamics, current thermodynamic models cannot capture this phenomenon. As a result, simulations performed on monoculture included an explicit constraint blocking net H_2 production.

2 Supporting Discussion

2.1 Testing and Parameterizing of the *i*Sfu648 Metabolic Model

The *i*Sfu648 model also has some important limitations. In particular, the *i*Sfu648 reconstruction does not distinguish between cytosolic, periplasmic, and membrane-bound versions of electron transport complexes (such as H_2ase). For example, the fumarate reductase and succinate dehydrogenase complexes are membrane-bound, and menaquinol should transfer electrons to a membrane-bound or periplasmic enzyme complex. However, the *i*Sfu648 reconstruction does not differentiate between differently-localized versions of the same complex. As a consequence, the *i*Sfu648 model's predicted electron transport mechanisms may be simpler than occur *in vivo*.

In addition, by using the standard transformed Gibbs free energy of reaction ($\Delta_r G^{'0}$) as a basis for thermodynamic calculations, the *i*Sfu648 model contained too much network flexibility, as it did not account for thermodynamic interconnectivity arising from shared metabolites. Thermodynamic interconnectivity can be captured using the standard transformed Gibbs free energy of molecules or groups directly when modeling thermodynamics, as well as through lumping constraints. Enumerating all such constraints which could be applied to a network is computationally infeasible, but it may be possible to identify important linear combinations of reactions which would benefit from a lumping constraint.

3 Supporting Methods

3.1 Reconstruction and Parameterization of the *i*Mhu428 Metabolic Model

The *i*Mhu428 reconstruction of *M. hungatei* was built from the *i*MB745 reconstruction of *M. acetivorans* [41], the newest methanogen reconstruction available at the time this work began. A preliminary draft reconstruction was built using the RAVEN Toolbox [42], but, the reconstruction contained less than 200 genes, or 27% of the genes in the *i*MB745 reconstruction (S2 Dataset in the Supporting Material). Instead of performing extensive gapfilling, all 756 reactions from the *i*MB745 *M. acetivorans* reconstruction were copied into the *i*Mhu428 reconstruction, with modifications to reflect key metabolic features of *M. hungatei*.

Because *M. hungatei* can only utilize acetate and CO_2 [55], reactions which enabled growth on CO and methylated carbon sources were removed. In addition, the oxidative arm of the TCA cycle was removed, and the reductive arm was added [71]. The methanogenesis pathway was also replaced [7].

The *i*Mhu428 reconstruction was then converted to a thermodynamic model, as described in “Metabolite Structures and Molecular Formulas” and “Thermodynamics-Based Metabolic Flux Analysis.” On defined minimal media (Table S1 in S1 Dataset in the Supporting Material), TMFA predicted a no-growth phenotype, due to the inability of the *i*Mhu428 model to oxidize H_2S to SO_3^{2-} via sulfite reductase, a necessary reaction for biomass production. The estimated transformed Gibbs free energy of reaction ($\Delta_r G^{'}$) for this reaction was between 55.6 kJ/mol and 284 kJ/mol, indicating that the reaction could not proceed in the forward direction required for growth. However, replacing the coenzyme F_{420} with a generic ferredoxin enabled sulfite reductase to proceed in the required direction, though the group contribution method could not provide an estimate of the standard transformed Gibbs free energy of reaction ($\Delta_r G^{'0}$). Because sulfur metabolism in methanogens remains poorly understood [66], this difference in cofactor utilization between *M. acetivorans* and *M. hungatei* seems possible.

Substrate uptake rates (SURs), and growth- (GAM) and non-growth-associated (NGAM) maintenance requirements for the *i*Mhu428 model of *M. hungatei* were estimated using experimental data. NGAM represents the amount of energy spent to maintain the cell (i.e., maintenance energy), while GAM represents energy spent on growth-related functions (e.g., protein synthesis). In genome-scale models, these ATP requirements are usually expressed using ATP hydrolysis reactions: in the case of NGAM, the ATP hydrolysis reaction is constrained to some lower bound, and in the case of GAM, an ATP hydrolysis term is added to the biomass equation.

The value of the NGAM parameter was found by maximizing ATP hydrolysis at the reported maintenance cost as measured in terms of CO_2 uptake [53]. The value of the GAM parameter is typically obtained by plotting a series of substrate uptake and growth rates taken from chemostat experiments [72], but no such data were available for *M. hungatei*. However, studies have reported the growth rate of *M. hungatei* on CO_2 [51], and its yield per mol methane ($Y_{X/P}$) [72]. From these data, the following iterative procedure was used to simultaneously estimate SUR_{CO2} and GAM:

1. Select an initial estimate for the GAM.
2. Identify the SUR_{CO2} for which the observed growth rate and model-predicted maximum growth rates agree.
3. Calculate the *in silico* CH_4 production rate at the model-predicted maximum growth rate and compute $Y_{X/P}$.
4. Adjust the value of the GAM upwards or downwards until $Y_{X/P}$ at the model-predicted maximum growth agrees with the experimental measurement. Changing the GAM will change the model-predicted maximum growth. Thus,
5. Repeat steps 2 to 4 until the model-predicted maximum growth and corresponding $Y_{X/P}$ agree with the experimental measurement.

An identical procedure was followed to compute the SUR for growth on formate, assuming the same NGAM and GAM as for growth on CO_2. For the *i*Mhu428 model, the NGAM was estimated to be 0.6 mmol ATP/gDW/day, GAM was estimated to be 47 mmol ATP/gDW, SUR_{CO2} was estimated to be 75.7 mmol/gDW/day, and $SUR_{formate}$ was estimated to be 955 mmol/gDW/day.

The final *i*Mhu428 reconstruction contains 806 reactions, 273 genes (associated with 285 reactions), 706 metabolites. Of the 273 genes, 196 were added based on sequence homology, and 77 were added manually. The *i*Mhu428 reconstruction is available in S2 Dataset and S3 Dataset, in Excel and SBML formats, respectively.

3.2 Reconstruction and Parameterization of the *i*Sfu648 Metabolic Model

The *i*Sfu648 reconstruction of *S. fumaroxidans* was built using the RAVEN Toolbox [42], which uses sequence orthology to construct draft reconstructions from the proteins and reactions in the KEGG database. Briefly, the RAVEN Toolbox uses protein homology to identify the KEGG Orthology (KO) ID, which best matches each gene. The reactions and genes corresponding to that KO ID are then imported into the *i*Sfu648 reconstruction. Sequence homology was computed using default parameters: e-value < 10^{-30}, alignment length > 200 nucleotides, sequence similarity > 40%. The resulting draft reconstruction was manually refined following recommendations given in a recent review [73]. While the *i*Sfu648 reconstruction was built using the RAVEN Toolbox, curation and refinement occurred in the GAMS modeling environment.

3.2.1 Curation of Draft *i*Sfu648 Model

First, non-metabolic reactions were removed from the draft reconstruction (primarily those involved in tRNA charging). Next, all generic metabolites (e.g., acceptor) were identified and replaced with specific metabolites (e.g., NAD). Then, all reactions in the *i*Sfu648 reconstruction were mass- and charge-balanced, with a generic ferredoxin molecule used for charge balancing if atomic balancing was insufficient to do so.

In addition, all gene-protein-reaction (GPR) relationships were evaluated. Instead of providing detailed GPRs, the RAVEN Toolbox generates lists of genes associated with each reaction, and requires users to define the detailed GPR structure themselves.

First, gene annotations were examined to ensure that they matched the function of the associated reactions. This was particularly problematic in the case of one-to-many relationships, in which a single protein can carry out multiple reactions. For each gene which matches a particular KO group, the RAVEN Toolbox associates all

reactions in that group with the gene. Many such reactions were removed, due to a low likelihood of physiological relevance. In addition, 80 of the reactions contained in the final *i*Sfu648 reconstruction had their GPRs adjusted through the removal of one or more genes, and 16 had their GPRs replaced entirely (approximately 11% of all reactions). The draft reconstruction contained a number of hypothetical genes, and both these genes and their associated reactions were removed from the *i*Sfu648 reconstruction.

The logical structures of the GPRs provided by the RAVEN Toolbox were also determined. In addition to simple associations, in which a single gene encodes a single enzyme, GPRs can take the form of isozymes, in which multiple genes encode distinct proteins carrying out the same function, and multimeric protein complexes, in which multiple genes encoding distinct protein subunits come together to form an active enzyme. Genes annotated as separate subunits of a complex were given an 'AND' relationship, while genes with no such annotation were given an 'OR' relationship. Of the reactions retained in the final *i*Sfu648 reconstruction, approximately 32% of them required manual curation of the logical relationships among the subunits and isozymes.

All told, the final *i*Sfu648 reconstruction retained 717 of 859 reactions (83%) and 556 of 720 genes (77%) from the draft reconstruction, and around 50% of the GPRs required some level of curation.

Finally, the RAVEN Toolbox does not provide reaction direction information, instead assuming reactions are bidirectional in the absence of any specifying information. Reaction directions for the *i*Sfu648 reconstruction were computed using group contribution, as described in the TMFA-LP section.

3.2.2 Manual Curation of Energy Conservation Mechanisms

S. fumaroxidans contains a wide variety of hydrogenases, dehydrogenases, and other enzyme complexes involved in electron transport [8,9,48–51]. A brief description of each reaction and justification for each annotation are given below. These reactions were incorporated into the final *i*Sfu648 reconstruction, replacing draft reconstruction content where it disagreed with experimental evidence.

3.2.2.1 Hydrogenases and Formate Dehydrogenases

S. fumaroxidans contains a large number of gene clusters encoding hydrogenases and formate hydrogenases, which we annotate as follows:

The gene cluster Sfum_0844-0846 encodes for a bifurcating [FeFe]-hydrogenase [8]. In *Thermotoga maratima*, this complex has been shown to simultaneously use electrons from NADH and reduced ferredoxin to produce H_2 [74], a process known as confurcation. A similar function has been proposed for a formate dehydrogenase (Sfum_2703-2707), and a [NiFe]-hydrogenase (Sfum_2713-2716) [8]. Experimental evidence supports these annotations [51], with the [NiFe]-hydrogenase having formate dehydrogenase activity. The reactions associated with these three complexes are described as "confurcating" H_2ases and FDHases in our model.

The gene clusters Sfum_0030-0031 and Sfum_1273-1275 are annotated as cytosolic formate hydrogenases, and the clusters Sfum_2952-2953 and Sfum_2220-2222 as cytosolic hydrogenases [8,48]. It has been proposed that these complexes are involved in succinate oxidation, where they receive electrons from b- and c-type cytochromes [8,48]. The cytochromes would in turn receive electrons from the

menaquinone pool, as has been observed in *Desulfovibrio vulgaris* [75]. However, group contribution methods cannot compute free energies for cytochromes and cytochrome-containing reactions, so we have chosen to lump the two steps into a single reaction, in which electrons are transferred directly from menaquinol. We describe these reactions as “cytochrome mediated” to emphasize the role cytochromes play in this process.

Finally, the genome of *S. fumaroxidans* contains clusters encoding for two cytoplasmic [NiFeSe]-hydrogenases (Sfum_3535-3537 and Sfum_3954-3956), and a cytoplasmic formate dehydrogenase (Sfum_3509-3511) [8,51]. Experimental evidence suggests these complexes are induced to reoxidize ferredoxin [51], so we have given these reactions ferredoxin reducing H_2ases and FDHase activity, respectively.

3.2.2.2 Formate-Hydrogen Lyase

The gene cluster Sfum_1791-1796 shows similar to those coding for cytoplasmic-oriented membrane proteins which interconvert hydrogen and CO_2 to formate [51], collectively referred to as formate-hydrogen lyase activity. Specifically, Sfum_1795-1796 show similarity to *fhlF*, and Sfum_1791 shows similarity to *fhlH*. We have assigned the entire cluster a formate-hydrogen lyase activity.

3.2.2.3 RNF Complex

The gene cluster Sfum_2694-2699 encodes for an RNF cluster [8,51], which has been propose to reoxidize NADH by ferredoxin reduction via the use of a proton motive force [8,51]. This proposal is consistent with the biochemistry of the RNF complex in a variety of prokaryotes [76].

3.2.2.4 Succinate Dehydrogenase

The gene cluster *sdhABC* (Sfum_1998-2000) codes for two cytoplasmic subunits (*sdhA* and *sdhB*) and a trans-membrane subunit containing heme (*sdhC*) which are similar to the succinate dehydrogenase subunits of *Bacillus subtilis* [48]. In addition, *S. fumaroxidans* contains menaquinones [37]. Oxidation of succinate to fumarate via menaquinone is an endergonic process ($\Delta_r G'^0 \approx$ 60 kJ/mol by our estimate). In *B. subtilis* and sulfate-reducing bacteria [77,78], the energy of a proton gradient is used to move electrons energetically “uphill” via a reverse electon transfer mechanism involving heme groups.

3.2.2.5 Fumarate Reductase

The gene cluster *frdABEF* (Sfum_4092-4095) encodes a fumarate reductase [48] which is believed to function similarly to that of *Wolinella succinogenes* [8,79]. In this bacterium, the transmembrane proton gradient is generated by hydrogen or formate oxidation coupled to fumarate reduction via cytochromes and a menaquinone loop [80]. While the proton translocation is believed to occur via the hydrogenases and formate dehydrogenases, the energy comes from fumarate reduction. Furthermore, our thermodynamic model predicts proton translocation via the H2ase or FDH to be impossible. For these reasons, we have modeled proton translocation as part of the fumarate reductase reaction.

3.2.2.6 NADH Dehydrogenase

The *S. fumaroxidans* genome contains two NADH dehydrogenase gene clusters (Sfum_0199-0209 and Sfum_1935-1943). We assigned the former to a NADH dehydrogenase using menaquinone without proton transport, and the latter to a NADH dehydrogenase using menaquinone with proton transport.

3.2.2.7 ATP Synthase

The endergonic oxidation of succinate to fumarate requires a proton gradient to function. Thermodynamic calculations suggest that about 0.67 mol ATP has to be invested to make this reaction energetically possible at conditions maintained by methanogens in a syntrophic community [5]. Because the succinate dehydrogenase reaction in the *i*Sfu648 model pumps two protons, the ATP synthase reaction is assumed to pump three protons.

3.2.3 Manual Curation of Carbon Transfer Mechanisms

S. fumaroxidans metabolizes carbon in five well-defined ways, as described in Table 1. The steps of each pathway have been determined by ^{13}C-NMR experiments [36,52], and a recent genomic survey identified the genes associated with each enzyme [48]. These pathways were incorporated into the final *i*Sfu648 reconstruction, replacing draft reconstruction content where it disagreed with experimental evidence.

3.2.4 Experimental Justification for Theoretical Flux Distributions

Experimental studies have elucidated six growth modes for *S. fumaroxidans*: five in monoculture and one in coculture with *M. hungatei* (Table 2) [36,48,52]. Brief descriptions of the three growth modes examined in this work are presented below.

3.2.4.1 Monoculture Growth on Fumarate

^{13}C labeling studies reveal that one mole of fumarate gets fully oxidized to CO_2 for every six moles of fumarate which get reduced to succinate [48,52]:

$$7 \text{ fumarate} \rightarrow 6 \text{ succinate} + 4\ CO_2 \qquad \text{(S9)}$$

Enzymatic assays revealed that oxidiation of fumarate to CO_2 generates six reducing equivalents (NADH or reduced ferredoxin) [48,52], while the reduction of fumarate to succinate is driven by menaquinol [48]. Based on the results of gene expression assays [51], we propose the following mechanism for the transfer of electrons from NADH and reduced ferredoxin to menaquinol. High levels of NADH from fumarate oxidation induce RNF, which reoxidizes NADH by ferredoxin reduction. In turn, ferredoxin induces expression of the ferredoxin-oxidizing formate dehydrogenase and hydrogenase, which transfer electrons from ferredoxin to protons, generating H_2. The cytosolic hydrogenase then transfers electrons from H_2 to menaquinone, generating menaquinol. Reduction of fumarate to succinate generates the proton motive force (PMF) responsible for driving RNF and producing ATP.

3.2.4.2 Monoculture Growth on Fumarate and Propionate

^{13}C labeling studies reveal that one mole of propionate gets oxidized to succinate, while one mole of fumarate gets oxidized to acetate and CO_2. An additional two moles of fumarate get reduced to succinate [36,48]:

$$\text{propionate} + 3 \text{ fumarate} \rightarrow \text{acetate} + CO_2 + 3 \text{ succinate} \qquad \text{(S10)}$$

Based on the results of gene expression assays [51], the following mechanism for energy conservation has been proposed. The oxidation of fumarate to acetate and CO_2 produces one mole NADH and one mole reduced ferredoxin. The confurcating hydrogenase couples NADH and ferredoxin oxidation with H_2 production. The cytosolic hydrogenase then transfers electrons from H_2 to menaquinone, generating menaquinol. Reduction of fumarate to succinate generates the proton motive force (PMF) responsible for ATP production.

3.2.4.3 Coculture Growth on Propionate

^{13}C labeling studies reveal that propionate gets oxidized to fumarate and CO_2 via the methylmalonyl-CoA pathway [48,52].

$$\text{propionate} \rightarrow \text{acetate} + CO_2 + 2\ H_2 \qquad \text{(S11)}$$

Based on the results of gene expression assays [51], the following mechanism for energy conservation has been proposed. A single ATP molecule is generated during the oxidation of propionate to succinate. This ATP molecule is hydrolyzed to generate the PMF necessary for the endergonic oxidation of succinate to fumarate. This oxidation generates menaquinol; the cytosolic hydrogenase then transfers electrons from menaquinol to two protons, generating H_2. The oxidation of fumarate to acetate and CO_2 produces one mole NADH and one mole reduced ferredoxin. The confurcating hydrogenase couples NADH and ferredoxin re-oxidation with H_2 production.

3.2.5 Regulatory Constraints

The expression levels of the enzymes described in 3.2.2 have been shown to vary across conditions [51]. We developed the following regulatory constraints to ensure the proper energy conservation mechanisms were active under each condition. These constraints are justified below, and are summarized in Table S3 in S1 Dataset of the Supporting Materials.

3.2.5.1 Confurcating Hydrogenases and Formate Dehydrogenases

Gene expression evidence indicates these reactions are expressed at statistically significant higher levels during coculture growth, and in monoculture growth with fumarate and propionate. Thus, we allow these reactions to carry flux only under those conditions.

3.2.5.2 RNF Complex and Ferredoxin-Oxidizing Hydrogenases and Formate Dehydrogenases

Experimental evidence suggests that high NADH levels may induce transcription of genes encoding for the RNF complex, which couples NADH oxidation to ferredoxin reduction [51]. The ferredoxin-oxidizing hydrogenases and formate dehydrogenases then reoxidize the ferredoxin [51]. Because growth on fumarate in monoculture generates significantly more NADH than other growth modes, we allow these reactions to carry flux only under that condition.

3.2.5.3 Cytosolic Hydrogenases and Formate Dehydrogenases

In the conceptual model of energy conservation in S. fumaroxidans, the cytosolic hydrogenases (Sfum_0030-0031 and Sfum_1273-1275) and formate dehydrogenases (Sfum_2952-2953 and Sfum_2220-2222) are involved in succinate oxidation and fumarate reduction, where they receive electrons from the menaquinone pool. We

introduced flux coupling constraints to ensure that electrons from succinate oxidation and fumarate reduction are transferred strictly to the cytosolic hydrogenases and formate dehydrogenases.

3.2.5.4 NADH dehydrogenases.

It is unknown what, if any, role the two NADH dehydrogenase gene clusters play in energy conservation. To the best of our knowledge, no conceptual models of energy conservation in *S. fumaroxidans* predict a role for the NADH dehydrogenases, and their expression levels were not measured in the single gene expression study we were able to find [51]. In light of this, we chose to constrain the two NADH dehydrogenase reactions to carry zero flux across all conditions.

3.2.5.5 Fumarate Reductase and Succinate Dehydrogenase

Fumarate reductase is predicted to be active only during growth in monoculture, and to exclusively reduce fumarate to succinate. Succinate dehydrogenase is predicted to be active only during growth in coculture, and to exclusively oxidize succinate to fumarate. Thus, these two reactions were constrained as appropriate during each simulation condition.

3.2.6 Development and Gapfilling of Biomass Equation

The RAVEN Toolbox does not provide users with a biomass equation; they must instead construct one manually. Unfortunately, the dry cell weight biomass composition of *S. fumaroxidans* has not yet been described. In this work, a template biomass equation (proposed by the developers of the Model SEED [81]) was used as a scaffold to construct the biomass equation based on data from related organisms.

The weight fractions of major macromolecule classes (proteins, DNA, etc) were taken from *Geobacter sulfurreducens*, a deltaproteobacterium which has been extensively studied and modeled [13,82]. (*S. fumaroxidans* is also a deltaproteobacterium). Mole fractions of individual macromolecules were obtained as follows:

- amino acids, from the *i*AF1260 model of *E. coli* [83];
- DNA and RNA, from the *S. fumaroxidans* genome sequence [48];
- lipids and carbohydrates, from *G. sulfurreducens* [84];
- cell wall, cofactors, and small molecules, from the Model SEED template.

Additional details can be found in Table S5 of S1 Dataset in the Supporting Materials.

The *i*Sfu648 model was unable to sustain flux through this biomass equation under any known growth condition. Individual biomass precursors which the *i*Sfu648 model could not synthesize were identified, and SMILEY was used to identify those reaction additions which would enable biomass growth [85]. Manual curation was used to identify reactions necessary for lipid, cell wall, and carbohydrate biosynthesis. Where possible, those solutions for which genomic evidence could be found were selected. In the case of many cofactors, no genomic evidence was found for any solution. These cofactors were eliminated from the biomass equation.

SMILEY is a mixed-integer programming approach for model refinement, which calculates the minimum number of reactions from a universal reaction database which must be added to a reconstruction to enable cellular growth. For this study, the universal reaction database was a manually-curated subset of version 57 of the KEGG

database, available through the BioWebDB Consortium (http://www.biowebdb.org). During curation, all reactions which met any of the following criteria were removed:

- contained an elongation;
- contained the same metabolite on both sides;
- contained compounds in the KEGG glycan database;
- contained a compound with an R group;
- contained a compound without a formula.

For reactions containing a generic acceptor molecule, two separate versions of the reaction were created, one using NAD as an acceptor, and one using NADP. Additionally, any reaction which could not be balanced through the addition of protons or ferredoxins was removed. Reaction directions for the gapfilling database were computed using group contribution, as described in the TMFA-LP section of Methods. Metabolite formulas and molecular structure files (molfiles) were obtained directly from KEGG. The curated database contains over 6000 reactions and represents approximately 70% of the original KEGG version 57 database. The curated database is provided in S6 Dataset and S7 Dataset, in Excel and SBML formats, respectively.

3.2.7 Gapfilling of Blocked Reactions

Flux Variability Analysis (FVA) [86] was used to identify reactions which were blocked under one or more growth conditions. SMILEY was used to identify gapfilling solutions for these blocked reactions, and those reactions with genomic evidence were added to the *i*Sfu648 model.

3.2.8 Conversion to a Thermodynamic Model

The *i*Sfu648 reconstruction was then converted to a thermodynamic model, as described in "Metabolite Structures and Molecular Formulas" and "Thermodynamics-Based Metabolic Flux Analysis." On defined minimal media (Table S1 in S1 Dataset in the Supporting Material), TMFA predicted a no-growth phenotype, most likely because biosynthetic routes which were feasible under TMFA-LP may not be feasible under TMFA [34]. Once again, SMILEY identified those biomass precursors which the *i*Sfu648 model could not synthesize, and solutions which would enable biomass growth under TMFA were added to the *i*Sfu648 reconstruction.

The final *i*Sfu648 reconstruction contains 874 reactions, 648 genes (associated with 770 reactions), 893 metabolites. Considering the entire content of the final *i*Sfu648 reconstruction, reactions and genes contained in the draft reconstruction make up 82% and 86% of the total reaction and gene content, respectively. The *i*Sfu648 reconstruction is available in S4 Dataset and S5 Dataset, in Excel and SBML formats, respectively.

3.2.9 *i*Sfu648 Model Parameterization

Substrate uptake rates (SURs), and growth- (GAM) and non-growth-associated (NGAM) maintenance requirements for the *i*Sfu648 model of *S. fumaroxidans* were estimated using experimental data.

The value of the NGAM parameter was found by maximizing ATP hydrolysis at the reported maintenance cost as measured in terms of propionate uptake [53].

The value of the GAM parameter is typically obtained by plotting a series of substrate uptake and growth rates taken from chemostat experiments [72], but no such data were available for *S. fumaxoridans*. However, studies have reported the growth rate of *S. fumaxoridans* on a variety of substrates [51], and its yield per mol propionate ($Y_{X/S}$) [72]. From these data, the following iterative procedure was used to simultaneously estimate $SUR_{propionate}$ and GAM:

1. Select an initial estimate for the GAM.
2. Identify the $SUR_{propionate}$ for which the observed growth rate and model-predicted maximum growth rates agree.
3. Adjust the value of the GAM upwards or downwards until $Y_{X/P}$ at the model-predicted maximum growth agrees with the experimental measurement. Changing the GAM will change the model-predicted maximum growth. Thus,
4. Repeat steps 2 to 3 until the model-predicted maximum growth and corresponding $Y_{X/P}$ agree with the experimental measurement.

Step 2 was used to compute the SUR for growth on fumarate, assuming the same NGAM and GAM as for growth on propionate. For the *i*Sfu648 model, the NGAM was estimated to be 3.36 mmol ATP/gDW/day, GAM was estimated to be 22.8 mmol ATP/gDW, $SUR_{propionate}$ was estimated to be 37.7 mmol/gDW/day, and $SUR_{fumarate}$ was estimated to be 27.6 mmol/gDW/day.

3.3 Metabolite Structures and Molecular Formulas

To estimate Gibbs free energies, molecular structure files (molfiles) for metabolites in the reconstructions were obtained from KEGG (*i*Mhu428 and *i*Sfu648) or manually constructed (*i*Mhu428). For the *i*Mhu428 reconstruction, molfiles were obtained for 94% of the metabolites, enabling the calculation of the standard transformed Gibbs free energy of reaction ($\Delta_r G^{'0}$) for 83% of the reactions. For the *i*Sfu648 reconstruction, molfiles were obtained for 100% of the metabolites, enabling the calculation of $\Delta_r G^{'0}$ for 84% of the reactions.

All metabolites were then converted to their predominant ionic species (pseudoisomer) at biochemical standard state: pH 7, zero ionic strength, and temperature 298K. The major pseudoisomeric form of each molecule was determined using pKa estimation software (Marvin pKa plug-in, version 5.11.4, ChemAxon, Budapest, Hungary). Finally, all reactions in the reconstructions were mass- and charge-balanced using the new metabolite formulas. Supplemental Files “S1 File” and “S2 File” contain molfiles for all compounds in the *i*Mhu428 and *i*Sfu648 reconstructions, respectively.

3.4 Thermodynamics-Based Metabolic Flux Analysis (TMFA)

Flux-balance analysis (FBA) [27] is a constraint-based technique for predicting the state of a metabolic network consistent with physiochemical principles. FBA identifies a flux distribution which maximizes cellular growth (or some other objective function), subject to steady-state mass-balance and enzyme capacity constraints.

Specifically, given a stoichiometric matrix S and a set of reactions J, FBA seeks a steady-state flux distribution v maximizing the flux through the biomass reaction (v_{BM}),

while also satisfying mass-balance and enzyme capacity constraints for individual reactions, *j*:

$$\mathbf{max} \quad v_{BM} \tag{S12}$$

$$\mathbf{s.t.} \quad S \cdot v = 0 \tag{S13}$$

$$v_{\min} \le v_j \le v_{\max} \qquad \forall j \in J \tag{S14}$$

Enzyme capacities, v_{min} and v_{max}, should be set on the basis of available evidence, such as thermodynamic irreversibility. Fluxes are typically constrained to v_{max} = 1000 mmol/gDW/day and v_{min} = −1000 mmol/gDW/day, except for measured fluxes (e.g., carbon uptake rates). Uptake rates used in the simulations are given in Table S1 in S1 Dataset of the Supporting Material.

Thermodynamics-Based Metabolic Flux Analysis (TMFA, [33,34]) extends FBA via the introduction of thermodynamic constraints. TMFA assumes no *a priori* evidence for enzyme directionality, instead enforcing the second law of thermodynamics, which states that the transformed Gibbs free energy of reaction ($\Delta_r G'$) and flux (*v*) have opposite signs:

$$v_j \cdot \Delta_r G_j^{'} < 0 \tag{S15}$$

This nonlinear constraint is converted to a mixed-integer constraint as described previously [34].

The $\Delta_r G^{'}$ of a reaction is in turn a function of the standard transformed Gibbs free energy of reaction ($\Delta_r G^{'0}$) and the concentrations (x_i) of those metabolites participating in the reaction:

$$\Delta_r G_j^{'} = \Delta_r G_j^{'0} + RT \sum_i S_{i,j} \ln(x_i) \tag{S16}$$

where *R* is the gas constant, and *T* is the temperature. In the absence of specific information, metabolite concentrations are constrained to global bounds of 0.01 mM to 20 mM. Specific metabolite concentrations were imposed for water, protons, and compounds in the growth media, as indicated in Table S2 in S1 Dataset of the Supporting Material.

Due to a paucity of experimental data, group contribution methods [61,87–89] are used to provide estimates and uncertainties of $\Delta_r G^{'0}$ for reactions. Estimates ($\Delta_r G_{j,est}^{'0}$) and uncertainties ($SE_{\Delta_r G_{j,est}^{'0}}$) of $\Delta_r G^{'0}$ for the reactions in the reconstructions were obtained using a software implementation of the latest group contribution method for biological systems, which the authors refer to as component contribution [61]. The implementation is available via the von Bertalanffy 2.0 add-on to the COBRA Toolbox [62].

The GCM method returns estimates of $\Delta_r G^{'0}$ for each reaction, given the pKa values for all compounds in the reconstruction, and information on temperature (T), pH, ionic strength (I) and electrical potential (Φ) in each cellular compartment. pKa values were calculated using pKa estimation software (Marvin pKa plug-in, version 5.11.4, ChemAxon, Budapest, Hungary) from molecular structure files (molfiles).

Thermodynamic parameters (T, pH, I, and Φ), and $\Delta_r G^{'0}_{j,est}$ and $SE_{\Delta_r G^{'0}_{j,est}}$ values for each reconstruction are given in S2 Dataset (*i*Mhu428) and S4 Dataset (*i*Sfu648).

The standard transformed Gibbs free energy ($\Delta_r G^{'0}$) of each reaction was allowed to vary within its 95% confidence interval, as determined by the standard error (SE) reported by the GCM software:

$$\Delta_r G^{'0}_{j,est} - 2SE_{\Delta_r G^{'0}_{j,est}} \leq \Delta_r G^{'0}_j \leq \Delta_r G^{'0}_{j,est} + 2SE_{\Delta_r G^{'0}_{j,est}} \quad \text{(S17)}$$

If the GCM method is unable to obtain an estimate of $\Delta_r G^{'0}$ for a particular reaction, $\Delta_r G^{'0}$ was allowed to vary freely.

The GCM method estimated $\Delta_r G^{'0}$ for approximately 82% of the reactions in the *i*Mhu428 reconstruction, and approximately 84% of the reactions in the *i*Sfu648 reconstruction. For some reactions for which $\Delta_r G^{'0}$ could not be estimated, a lumping approach was used to constrain $\Delta_r G^{'}$, as described in "Thermodynamic Lumping."

Aggregating the above constraints gives the final formulation for TMFA:

$$\begin{array}{llll} \max & v_{BM} & & \\ \text{s.t.} & \text{FBA constraints, (S13) and (S14)} & \forall j \in J & \\ & \Delta_r G^{'} \text{ constraints, (S16) and (S17)} & \forall j \in J & \text{(TMFA)} \\ & \text{consistency constraints, (S15)} & \forall j \in J & \end{array}$$

3.5 TMFA and Thermodynamics of H_2 Production

When evaluating the thermodynamic feasibility of H_2 production, simulations with the *i*Sfu648 model were performed using estimates of Gibbs free energies of formation ($\Delta_f G^{'0}_{i,est}$) as a basis for thermodynamic calculations. This switch from $\Delta_r G^{'0}_{j,est}$ to $\Delta_f G^{'0}_{i,est}$ as basis for thermodynamic calculations is necessary to capture the thermodynamic interconnectivity (arising from shared metabolites) between the individual reactions which contribute to the overall metabolic transformation. For these simulations, the formulation in the previous section was extended as follows:

First, the standard transformed Gibbs free energy of reaction $\Delta_r G^{'0}$ was computed as a function of the standard Gibbs free energy of formation $\Delta_f G^{'0}$ of its constituent metabolites, plus adjustments ($\Delta_{pH} G^{'0}_r$ and $\Delta_\Psi G^{'0}_r$) due to changes in pH gradient (*ΔpH*) and electrochemical potential (*ΔΨ*) across the cell membrane:

$$\Delta_r G^{'0}_j = \sum_i S_{i,j} \Delta_f G^{'0}_i + \Delta_{pH} G^{'0}_r + \Delta_\Psi G^{'0}_r \quad \text{(S18)}$$

Values for $\Delta_{pH} G^{'0}_r$ and $\Delta_\Psi G^{'0}_r$ were computed by the GCM software, as described previously [33,34,61]. Estimates ($\Delta_f G^{'0}_{i,est}$) and uncertainties ($SE_{\Delta_f G^{'0}_{i,est}}$) of $\Delta_f G^{'0}$ for the metabolites in the reconstructions were obtained using the GCM software, and $\Delta_f G^{'0}_{i,est}$ and $SE_{\Delta_f G^{'0}_{i,est}}$ values are given in S4 Dataset.

The standard transformed Gibbs free energy ($\Delta_f G^{'0}$) of each metabolite was allowed to vary within its 95% confidence interval, as determined by the standard error (SE) reported by the GCM software:

$$\Delta_f G^{'0}_{i,est} - 2SE_{\Delta_f G^{'0}_{i,est}} \leq \Delta_f G^{'0}_{i} \leq \Delta_f G^{'0}_{i,est} + 2SE_{\Delta_f G^{'0}_{i,est}} \quad \text{(S19)}$$

If the GCM method is unable to obtain an estimate of $\Delta_f G^{'0}$ for a particular reaction, $\Delta_f G^{'0}$ was allowed to vary freely.

Aggregating these constraints with the formulation from the previous section gives the final formulation:

$$\begin{array}{llll} \textbf{max} & v_{BM} & & \\ \textbf{s.t.} & \text{FBA constraints, (S13) and (S14)} & \forall j \in J & \\ & \Delta_r G^{'} \text{ constraints, (S16) and (S17)} & \forall j \in J & \text{(TMFA-dGf)} \\ & \Delta_r G^{'0} \text{ constraints, (S19) and (S23)} & \forall j \in J & \\ & \text{consistency constraints, (S15)} & \forall j \in J & \end{array}$$

After each H_2-producing mode was identified, the overall transformation was introduced as a new reaction to the *i*Sfu648 model. To block flux through the reaction, a constraint was sought which would ensure $\Delta_r G^{'} > 0$ for the reaction, thereby eliminating the erroneous H_2-producing phenotype. (We have previously referred to such constraints as phenotype-correction constraints [34].) That is,

$$\Delta_r G^{'0}_j + RT \sum_i S_{i,j} \ln(x_i) > 0 \quad \text{(S20)}$$

Then, as long as

$$\max RT \sum_i S_{i,j} \ln(x_i) > -\min \Delta_r G^{'0}_j \quad \text{(S21)}$$

adding a new constraint of the form

$$RT \sum_i S_{i,j} \ln(x_i) > -\min \Delta_r G^{'0}_j \quad \text{(S22)}$$

will ensure H_2 production via the identified mechanism is infeasible. Values of $\max RT \sum_i S_{i,j} \ln(x_i)$ and $\min \Delta_r G^{'0}_j$ were found by solving TMFA-dGf with the objective function of $\max RT \sum_i S_{i,j} \ln(x_i)$ or $\min \Delta_r G^{'0}_j$ as appropriate.

If such a constraint could be identified, it was added to the *i*Sfu648 model and new H_2-producing reactions were sought. The process was repeated until no additional H_2-productiong reactions could be found, or a phenotype-correction constraint could not be identified.

3.6 Thermodynamic Lumping

Due to limitations in the GCM [61], there were some reactions for which $\Delta_r G^{'0}$ could not be estimated. Among these reactions were the formate dehydrogenase and confuracting hydrogenase involved in H_2 production during syntophic growth. To understand why these reactions were predicted to be active under monoculture

conditions, a previously described lumping approach [34] was used to constrain the free energies of these reactions.

In this approach, reactions with unknown $\Delta_r G^{'0}$ are linearly combined into lumped representations for which $\Delta_r G^{'0}$ can be calculated. The set of lumped reactions is called J_L, and introduced into the S matrix as a subset of the set J. Constraints are derived which ensure both the lumped reactions and their constituent reactions remained thermodynamically consistent, irrespective of the value of $\Delta_r G^{'0}$ of the unknown reactions.

For lumped reactions, $\Delta_r G^{'}$ is calculated as described in (S16), with (S17) modified to allow $\Delta_r G^{'0}$ to vary freely. The transformed Gibbs free energy ($\Delta_r G^{'}$) of the lumped reactions is calculated from $\Delta_r G^{'}$ of their constituent reactions. To do this, parameters are defined such that $\alpha_{j,l}$ = 1 if reaction j (one of the reactions with unknown $\Delta_r G^{'0}$) combines in the forward direction to make up lumped reaction l, and defined $\alpha_{j,l}$ = −1 if reaction j combines in the reverse direction. The transformed Gibbs free energy ($\Delta_r G^{'}$) of the lumped reaction is then computed:

$$\Delta_r G_l^{'} = \sum_j \alpha_{j,l} \Delta_r G_j^{'} \qquad \forall l \in J_L \tag{S23}$$

This constraint ensures that the thermodynamics of the lumped reaction and its constituents are internally consistent, irrespective of the value of $\Delta_r G^{'0}$ of the unknown reactions. The complete list of lumped reactions J_L, and parameters α can be found in S4 Dataset in the Supporting Material.

3.7 Linear Programming Approximation of TMFA (TMFA-LP)

Because TMFA is a mixed-integer program, its use may be impractical under certain conditions. For example, TMFA cannot be used as the inner problem in a bilevel optimization, and extending TMFA to large networks (as in gapfilling) is computationally intensive. As a consequence, during model refinement TMFA-LP, rather than TMFA, is used as described previously [34]. In this approach, extreme values for $\Delta_r G^{'}$ are computed as given by (S16), assuming $\Delta_r G^{'0}$ falls within the 95% confidence interval given by (S17). If the predicted $\Delta_r G^{'}$ range for a reaction was entirely negative, the reaction is assumed to proceed only in the forward direction (i.e., v_{min} = 0 mmol/gDW/day); if the predicted $\Delta_r G^{'}$ range is entirely positive, the reaction is assumed to proceed only in the reverse direction (i.e., v_{max} = 0 mmol/gDW/day). Otherwise, the reaction is allowed proceed in both directions (i.e., v_{min} = -1000 mmol/gDW/day, v_{min} = 1000 mmol/gDW/day).

3.8 Parsimonious TMFA (pTMFA)

While FBA often assumes selective pressure for the fastest growing strains, other selective forces may shape an organism’s phenotype [90]. pFBA [54] is a constraint-based approach which assumes selective pressure not only for the fastest growing strains, but also for the lowest total flux through the network (a proxy for minimizing the

total mass of enzymes required to sustain optimal growth through the network). pTMFA uses the same assumptions as pFBA while implementing the thermodynamic constraints of TMFA.

pTMFA was implemented as a two-stage optimization process. In Stage 1, TMFA is solved as described previously, where growth rate is maximized. In the second stage, the total flux through the network is minimized, by decomposing each flux v into its non-negative forward and reverse components (v^+ and v^-), and summing over the flux components for all reactions in the network:

$$\min \quad \sum_j v_j^+ + v_j^- \qquad \text{(S24)}$$

$$\text{s.t.} \quad v = v^+ - v^- \qquad \text{(S25)}$$

$$v^+ \geq 0, \quad v^- \geq 0 \qquad \text{(S26)}$$

To ensure that the pTMFA solution is also optimal with respect to growth, the growth rate is fixed to the Stage 1 solution:

$$v_{BM} = \max \ \text{(TMFA)} \qquad \text{(S27)}$$

Aggregating the new constraints with those from TMFA gives pTMFA:

$$\begin{aligned}
\min \quad & \sum_j v_j^+ + v_j^- & & \\
\text{s.t.} \quad & \text{FBA constraints, (S13) and (S14)} & \forall j \in J & \\
& \Delta_r G' \text{ constraints, (S16) and (S17)} & \forall j \in J & \qquad \text{(pTMFA)} \\
& \text{consistency constraints, (S15)} & \forall j \in J & \\
& \text{flux decomposition constraints, (S25) and (S26)} & \forall j \in J & \\
& \text{optimal biomass given by TMFA, (S27)} & &
\end{aligned}$$

3.9 Community Formulation

For the coculture simulations, a community model which simulates growth in a continuous stirred-tank reactor (CSTR) was developed. The formulation takes into account the biomass concentrations X_n of each species, n. Conceptually, the formulation attempts to minimize the species-weighted total flux through the metabolic networks, subject to TMFA constraints for each species, and a mass balance around the entire reactor:

$$\begin{aligned}
\min \quad & \sum_n \left(X_n \sum_{j,n} \left(v_{j,n}^+ + v_{j,n}^- \right) \right) & & & \\
\text{s.t.} \quad & \text{FBA constraints, (S13) and (S14)} & \forall j \in J & \quad \forall n \in N & \\
& \Delta_r G' \text{ constraints, (S16) and (S17)} & \forall j \in J & \quad \forall n \in N & \qquad \text{(CSTR)} \\
& \text{consistency constraints, (S15)} & \forall j \in J & \quad \forall n \in N & \\
& \text{reactor balance, (S28)} & & & \\
& v_{BM,n} = D & & \quad \forall n \in N &
\end{aligned}$$

As described in Results and illustrated in Fig 3B, both species exchange metabolites with a shared pool of metabolites I. Each metabolite i_n is exported to (or imported from) the shared pool to species, n, via an exchange reaction j_n at flux $v_{j,n}$ (with

units of mmol/gDW/day). Likewise, each metabolite *i* has a net in (or out) flow from the reactor, denoted by rate F_i (note the units for F are mmol/L/day). Thus, each metabolite can flow into/out of the shared pool via $|N|+1$ reactions. Such tuples of reactions are indicated by the set $j_{n=1},\ldots, j_{n=|N|}, i$, with the set J_{Shared} containing all such tuples. The mass balance around the reactor can be then written:

$$\sum_n X_n v_{j,n} = F_i \qquad \forall (j_{n=1},\ldots,j_{n=|N|},i) \in J_{Shared} \tag{S28}$$

Realizing that the reactor operates at a fixed dilution rate (D) [91], the final constraint can be derived from a mass-balance around each species:

$$v_{BM,n} = D \qquad \forall n \in N \tag{S29}$$

As presented, this formulation is general and can be applied to a community with any number of species.

The use of thermodynamic feasibility constraints necessitates the use of concentrations as variables in the coculture formulation. Additional constraints enforce this consistency of metabolite concentrations. A set of tuples ($i_{n=1},\ldots,i_{n=|N|}, i$, called I_{Shared}) indicates those tuples of *in silico* metabolites corresponding to the same physical metabolite (e.g., CO_2 has an extracellular version for each strain, and a version in the shared pool, for a total of three versions). A constraint forces each version of a metabolite to have the same concentration, thus ensuring that a concentration constraint on metabolite *i* in the shared pool constrains the metabolite concentration in each model as well:

$$\ln(x_{i,n=1}) = \ldots = \ln(x_{i,n=|N|}) = \ln(x_i) \qquad \forall (i_{n=1},\ldots,i_{n=|N|},i) \in I_{Shared} \tag{S30}$$

Failing to do so may result in inconsistent thermodynamic predictions arising from a single metabolite having multiple concentrations.

Finally, metabolites known to be exported to (or imported from) the shared space by each species via diffusion are identified. For each such metabolite, a pair $(\hat{i}_n, \bar{i}_n)$ containing the extra- ($\hat{i}$) and intracellular ($\bar{i}$) versions of that metabolite gets defined, as do sets $I_{n,Out}$ and $I_{n,In}$ containing those pairs which diffuse out and in, respectively. Constraints on these metabolite concentrations ensure consistency with diffusion:

$$\begin{aligned} \ln(x_{\hat{i},n}) &< \ln(x_{\bar{i},n}) \qquad \forall (\hat{i}_n, \bar{i}_n) \in I_{n,Out} \\ \ln(x_{\hat{i},n}) &> \ln(x_{\bar{i},n}) \qquad \forall (\hat{i}_n, \bar{i}_n) \in I_{n,In} \end{aligned} \tag{S31}$$

The expanded coculture model formulation includes those constraints necessary for thermodynamic consistency across models.

$$\begin{array}{llll} \textbf{min} & \sum_n \left(X_n \sum_{j,n} \left(v^+_{j,n} + v^-_{j,n} \right) \right) & & \\ \textbf{s.t.} & \text{FBA constraints, (S13) and (S14)} \quad \forall j \in J & \forall n \in N & \\ & \Delta_r G' \text{ constraints, (S16) and (S17)} \quad \forall j \in J & \forall n \in N & \\ & \text{consistency constraints, (S15)} \quad \forall j \in J & \forall n \in N & \text{(CSTR)} \\ & \text{concentration consistency constraints, (S30)} & \forall n \in N & \\ & \text{diffusion constraints, (S31)} & \forall n \in N & \\ & \text{reactor balance, (S28)} & & \\ & v_{BM,n} = D & \forall n \in N & \end{array}$$

Definitions for the sets $I_{n,In}$, $I_{n,Out}$, and concentrations for metabolites in the shared pool are given in Table S2 of S1 Dataset of the Supporting Material.

This formulation is a mixed-integer non-linear program (MINLP): the thermodynamic consistency constraints (S4) are integer constraints, and the reactor balance constraint (S12) is nonlinear. To avoid solving the MINLP, the dilution rate D and biomass concentrations of X_n for all species were fixed, converting the MINLP into an easily-solvable MIP.

To explore the behavior of the community under a variety of operating conditions, the reactor dilution rate and the relative ratio of *M. hungatei* to *S. fumaroxidans* were systematically changed, while allowing unlimited propionate uptake by the reactor ($F_{\text{propionate}}$). Simulations were performed at dilution rates between 0 to 0.2 days^{-1} at an interval of 0.01 days^{-1}. The ratio of *M. hungatei* to *S. fumaroxidans* was varied from 0 to 1.6, with simulations performed at intervals of 0.1. The maximal dilution rate of 0.2 days^{-1} corresponds to the maximal growth rate of the slower-growing *S. fumaroxidans*.

3.10 Community Thermodynamic Variability Analysis

We performed community thermodynamic variability analysis (cTVA) to identify thermodynamically feasible metabolite concentration ranges for each metabolite in the coculture. Simulations were performed at dilution rates between 0 to 0.2 days^{-1} at an interval of 0.01 days^{-1}. The ratio of *M. hungatei* to *S. fumaroxidans* was varied from 0 to 1.6, with simulations performed at intervals of 0.1. Feasible metabolite concentration ranges for the coculture were obtained by maximizing (or minimizing) individual metabolite concentrations, subject to the constraints of the CSTR formulation. In addition, the species-weighted total flux through the metabolic network was constrained to the minimal value predicted by the CSTR formulation. That is, for each metabolite, the following optimization was performed:

$$
\begin{array}{lll}
\textbf{max (min)} & \ln(x_{i,n}) & \\
\textbf{s.t.} & \text{FBA constraints, (S13) and (S14)} \quad \forall j \in J & \forall n \in N \\
& \Delta_r G' \text{ constraints, (S16) and (S17)} \quad \forall j \in J & \forall n \in N \\
& \text{consistency constraints, (S15)} \quad \forall j \in J & \forall n \in N \\
& \text{concentration consistency constraints, (S30)} & \forall n \in N \\
& \text{diffusion constraints, (S31)} & \forall n \in N \\
& \text{reactor balance, (S28)} & \\
& D = \hat{D} & \forall n \in N \\
& \dfrac{X_{S.fum}}{X_{M.hun}} = \dfrac{\hat{X}_{S.fum}}{\hat{X}_{M.hun}} & \\
& \sum_n \left(X_n \sum_{j,n} \left(v^+_{j,n} + v^-_{j,n} \right) \right) \text{ given by solving (CSTR) for } \hat{D}, \hat{X}_{S.fum}, \hat{X}_{M.hun} &
\end{array}
\qquad \text{(cTVA)}
$$

3.11 Minimal Probabilistic Sets (MPS)

Under some conditions, thermodynamic feasibility constraints predict that a solution space contains undesired flexibility (e.g., the maximal predicted rate of ATP hydrolysis

exceeds the experimental observation). A previous study of thermodynamic constraints used probability to qualitatively constrain reaction directions and reduce network flexibility [68]. This approach calculated the probability that a reaction's $\Delta_r G'^0$ was negative. If the probability was greater (less) than 70% (30%), the reaction was constrained to the forward (reverse) direction. Otherwise, the reaction is assumed to be bidirectional.

This probabilistic approach was applied to reduce network flexibility in *M. hungatei*, but was found to reduce network flexibility too much, and phenotypes which were previously correct become incorrect (e.g., the model no longer predicted growth). To prevent this, an optimization procedure was developed to identify the smallest number of probabilistic (qualitative reaction direction) constraints needed to correct one or more phenotypes, while preserving one or more other phenotypes. The required formulation, Minimal Probabilistic Sets (MPS) maximizes network flexibility (as measured by the number of unconstrained reactions), with the probabilistic (qualitative reaction direction) constraints identified by subtraction. The formulation is defined as follows:

The sets $J_{probFwd}$ and $J_{probRev}$ consist of those reactions j in J which probability suggests should be constrained to the forward or reverse directions, respectively, while the set J_{Bidir} contains those reactions assumed to be directional. The binary variables y and z take a value of 0 if a reaction is to be constrained to the forward or reverse direction, respectively, and take a value of 1 otherwise. The appropriate constraints are:

$$\begin{aligned} y_j &= \{0,1\} & \forall j \in J_{probFwd} \\ z_j &= 1 & \forall j \in J_{probFwd} \end{aligned} \qquad \text{(S32)}$$

$$\begin{aligned} z_j &= \{0,1\} & \forall j \in J_{probRev} \\ y_j &= 1 & \forall j \in J_{probRev} \end{aligned} \qquad \text{(S33)}$$

$$y_j = z_j = 1 \qquad \forall j \in J_{Bidir} \qquad \text{(S34)}$$

The objective is to maximize the network flexibility (as measured by the number of unconstrained reactions), while maintaining consistency with each n of N phenotypes:

$$\mathbf{max} \quad \sum_{j,n} y_{j,n} + \sum_{j,n} z_{j,n} \qquad \text{(S35)}$$

$$\begin{aligned} \mathbf{s.t.} \quad & \text{phenotype constraints} & \forall n \in N \qquad & \text{(S36)} \\ & \text{computed cellular phenotype} & \forall n \in N \qquad & \text{(S37)} \\ & y_{j,n} = y_{j,\hat{n}} & \forall (n,\hat{n}) \in N \qquad & \\ & z_{j,n} = z_{j,\hat{n}} & \forall (n,\hat{n}) \in N \qquad & \text{(S38)} \end{aligned}$$

The final constraint (S38) ensures the same qualitative reaction direction constraints are applied to each computed cellular phenotype (S37).

Those reactions which require a qualitative reaction direction constraint can be easily identified, as the y or z variable associated with that reaction will have a value of 0. The desired phenotypes (S36) can be imposed as a variety of constraints, e.g.,

$$\begin{aligned} v_{BM} &\geq \varepsilon \\ v_{ATP} &= \gamma \end{aligned} \qquad \text{(S39)}$$

and may enforce phenotypes to be corrected (e.g., ATP gain) or maintained (e.g., cellular growth). Individual cellular phenotypes (S37) are computed by an inner problem,

which maximizes or minimizes some cellular objective, $v_{obj,}$ subject to mass-balance and qualitative reaction direction constraints under a particular media condition:

$$\begin{aligned} \textbf{max} \quad & v_{obj} && && \text{(S40)} \\ \textbf{s.t.} \quad & S \cdot v = 0 && && \text{(S41)} \\ & v_{\min} \le v_j \le v_{\max} && \forall j \in J && \text{(S42)} \\ & \text{media constraints} && && \text{(S43)} \\ & v_j \ge 0 && \forall j \in y_j = 0 && \text{(S44)} \\ & v_j \le 0 && \forall j \in z_j = 0 && \text{(S45)} \end{aligned}$$

The inner problem enforces the qualitative reaction direction (probabilistic) constraints (S44) and (S45) identified by the outer problem. Because thermodynamic feasibility constraints cannot be enforced directly in the inner problem, reactions are constrained using their TMFA-LP directions instead.

The final outer problem constraint (S38) ensures that the same qualitative reaction direction constraints are enforced on each inner problem. Aggregating all of the above constraints gives the final formulation for MPS:

$$\begin{aligned} \textbf{max} \quad & \sum_{j,n} y_{j,n} + \sum_{j,n} z_{j,n} && \\ \textbf{s.t.} \quad & \text{computed cellular phenotype(s), (S40) to (S45)} && \forall n \in N \\ & \text{phenotype constraint(s), (S39)} && \forall n \in N \qquad \text{(MPS)} \\ & \text{allowed probabilistic constraints, (S32) to (S34)} && \forall n \in N \\ & \text{consistency of probabilistic constraints, (S38)} && \forall n \in N \end{aligned}$$

For purposes of implementation, this bilevel problem is converted to a single-level problem via well-established techniques which rely on duality theory [92,93].

The formulation enables any number of phenotypes to be predicted by the inner problems, provided that a constraint for each is given in the outer problem. MPS was able to correct a number of phenotypes in the *i*Mhu428 and *i*Sfu648 models.

3.11.1 MPS for Validating *i*Mhu428: ATP Gain

Experimental evidence suggests that *M. hungatei* is able to generate 0.5 mol ATP per mol of CO_2 converted to CH_4 [7].[7]. A methanogenesis pathway which produces this ATP yield could be identified, but the *i*Mhu428 model predicted other, higher-yielding, ATP-generating mechanisms outside the methanogenesis pathway. MPS was used to identify qualitative reaction direction constraints which would eliminate these extra mechanisms, while simultaneously ensuring biomass growth:

$$\begin{aligned} \textbf{max} \quad & \sum_j y_j + \sum_j z_j && \\ \textbf{s.t.} \quad & \textbf{\textit{max}} \quad \text{ATP gain} && \\ & \textbf{\textit{s.t.}} \quad \text{FBA constraints, (S13) and (S14)} && \forall j \in J \\ & \qquad \text{carbon uptake via } CO_2 \text{ and acetate} && \qquad \text{(MPS)} \\ & \qquad \text{enforce probabilistic constraints, (S44) and (S45)} && \forall j \in J \\ & \text{ATP gain} = 0.5 && \\ & v_{BM} \ge 0.01 && \\ & \text{allowed probabilistic constraints, (S32) to (S34)} && \forall n \in N \end{aligned}$$

$$\text{consistency of probabilistic constraints, (S38)} \qquad \forall n \in N$$

MPS identified a set of 38 reactions to be constrained to a single direction (listed in Table S2 in S1 Dataset of the Supporting Material).

3.11.2 MPS for Validating *i*Mhu428: Biomass Growth

It has been observed that biomass and energy generation are independent and non-interacting in *M. hungatei*, with methanogenesis via CO_2 being the sole source of ATP, and acetate being the sole source of carbon in the biomass [55]. In contrast to experimental evidence, the *i*Mhu428 model predicts that *M. hungatei* can produce some biomass from CO_2 alone. MPS was used to resolve the discrepancy with two inner problems: the first inner problem tries to enforce a no growth phenotype on CO_2 alone, while the second inner problem tries to maintain growth on acetate alone:

$$
\begin{aligned}
\mathbf{max} \quad & \sum_{j,n} y_{j,n} + \sum_{j,n} z_{j,n} \\
\mathbf{s.t.} \quad & \boldsymbol{max} \quad v_{BM} \\
& \boldsymbol{s.t.} \quad \text{FBA constraints, (S13) and (S14)} \qquad \forall j \in J \\
& \qquad\quad \text{carbon uptake via } CO_2 \\
& \qquad\quad \text{enforce probabilistic constraints, (S44) and (S45)} \ \forall j \in J \\
& v_{BM} = 0 \\
& \boldsymbol{max} \quad v_{BM} \\
& \boldsymbol{s.t.} \quad \text{FBA constraints, (S13) and (S14)} \qquad \forall j \in J \\
& \qquad\quad \text{carbon uptake via acetate} \\
& \qquad\quad \text{enforce probabilistic constraints, (S44) and (S45)} \ \forall j \in J \\
& v_{BM} \geq 0.01 \\
& \text{allowed probabilistic constraints, (S32) to (S34)} \qquad \forall n \in N \\
& \text{consistency of probabilistic constraints, (S38)} \qquad \forall n \in N
\end{aligned}
\qquad \text{(MPS)}
$$

The problem proved infeasible, meaning that with the current *i*Mhu428 model topology ATP generation and biomass growth cannot be fully uncoupled.

3.11.3 MPS for Validating *i*Sfu648: Closed-Network ATP Cycles

Thermodynamic feasibility constraints dictate that solutions containing closed cycles (e.g., A → B → C → A) should be infeasible. In the absence of complete thermodynamic information, such cycles remain possible. MPS was used to identify qualitative reaction direction constraints which would eliminate closed-cycles which synthesize ATP, while simultaneously ensuring biomass growth:

$$
\begin{aligned}
\mathbf{max} \quad & \sum_{j,n} y_{j,n} + \sum_{j,n} z_{j,n} \\
\mathbf{s.t.} \quad & \boldsymbol{max} \quad v_{ATPM} \\
& \boldsymbol{s.t.} \quad \text{FBA constraints, (S13) and (S14)} \qquad \forall j \in J \\
& \qquad\quad \text{no uptake fluxes} \\
& \qquad\quad \text{enforce probabilistic constraints, (S44) and (S45)} \ \forall j \in J \\
& v_{ATPM} = 0 \\
& \boldsymbol{max} \quad v_{BM}
\end{aligned}
\qquad \text{(MPS)}
$$

$$
\begin{aligned}
&\qquad \textbf{s.t.} \quad \text{FBA constraints, (S13) and (S14)} && \forall j \in J \\
&\qquad\qquad\quad \text{growth in monoculture (or coculture)} \\
&\qquad\qquad\quad \text{enforce probabilistic constraints, (S44) and (S45)} && \forall j \in J \\
&\quad v_{BM} \geq 0.01 \\
&\quad \text{allowed probabilistic constraints, (S32) to (S34)} && \forall n \in N \\
&\quad \text{consistency of probabilistic constraints, (S38)} && \forall n \in N
\end{aligned}
$$

MPS identified a set of two reactions to be constrained to a single direction during monoculture growth, and one direction to be constrained during coculture growth (listed in Table S2 in S1 Dataset of the Supporting Material).

MPS was used also to identify a single set qualitative reaction direction constraints which would ensure growth in both monoculture and coculture simultaneously (formulation not shown). That problem was infeasible, indicating that distinct sets of qualitative reaction direction (probabilistic) constraints are required under the two conditions.

3.11.4 MPS for Validating *i*Sfu648: ATP Gain

In-silico simulations identified the theoretical maximum ATP yield for each growth mode of *S. fumaroxidans*. However, the *i*Sfu648 model enabled other mechanisms of ATP generation with a higher yield. MPS was used to identify qualitative reaction direction constraints which would eliminate these extra mechanisms, while simultaneously ensuring biomass growth:

$$
\begin{aligned}
&\textbf{max} \quad \sum_j y_j + \sum_j z_j \\
&\textbf{s.t.} \quad \textbf{max} \quad \text{ATP gain} \\
&\qquad\quad \textbf{s.t.} \quad \text{FBA constraints, (S13) and (S14)} && \forall j \in J \\
&\qquad\qquad\quad \text{appropriate growth mode uptake constraints} \\
&\qquad\qquad\quad \text{enforce probabilistic constraints, (S44) and (S45)} && \forall j \in J \\
&\quad \text{maximum ATP gain for that mode} \\
&\quad v_{BM} \geq 0.01 \\
&\quad \text{allowed probabilistic constraints, (S32) to (S34)} && \forall n \in N \\
&\quad \text{consistency of probabilistic constraints, (S38)} && \forall n \in N
\end{aligned}
\qquad \text{(MPS)}
$$

MPS identified a set of 38 reactions to be constrained to a single direction (listed in Table S2 in S1 Dataset of the Supporting Material).

3.12 Simulation Conditions

Simulations were performed for both individual models (*i*Sfu648 and *i*Mhu428) and the coculture model. Uptake fluxes for carbon and other nutrients are given in Table S1 in S1 Dataset of the Supporting Material. All simulations were performed using CPLEX 12 (IBM, Armonk, NY) accessed via the General Algebraic Modeling System, Version 23.9.5 (GAMS, GAMS Development Corporation, Washington, DC).

4 Supporting Figures

4.1 Fig S1

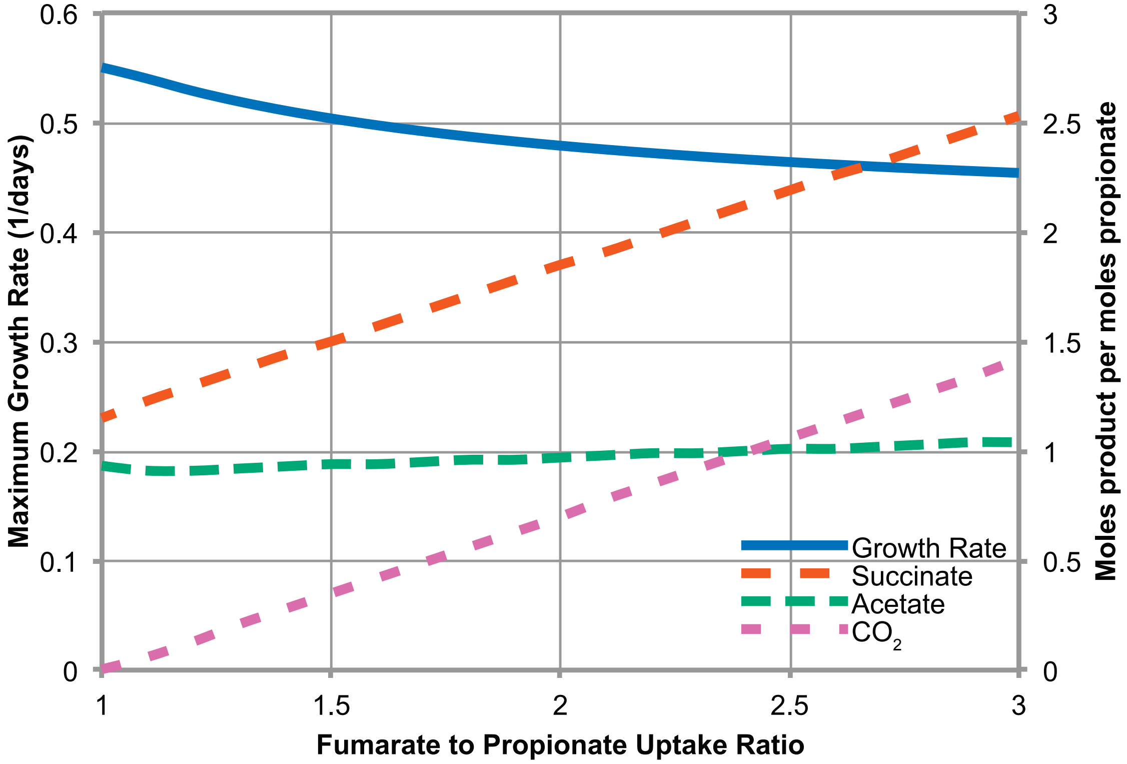

Growth rate and product stoichiometry of *S. fumaroxidans* in monoculture on fumarate with propionate. The fumarate to propionate uptake ratio was increased from one to three (the experimentally observed value) at intervals of 0.1. Simulations maximized growth rate, then minimized total enzyme cost (pTMFA). The experimentally observed product yields are three mol succinate per mol propionate; one mol acetate per mol propionate and one mol CO_2 per mol propionate.

4.2 Fig S2

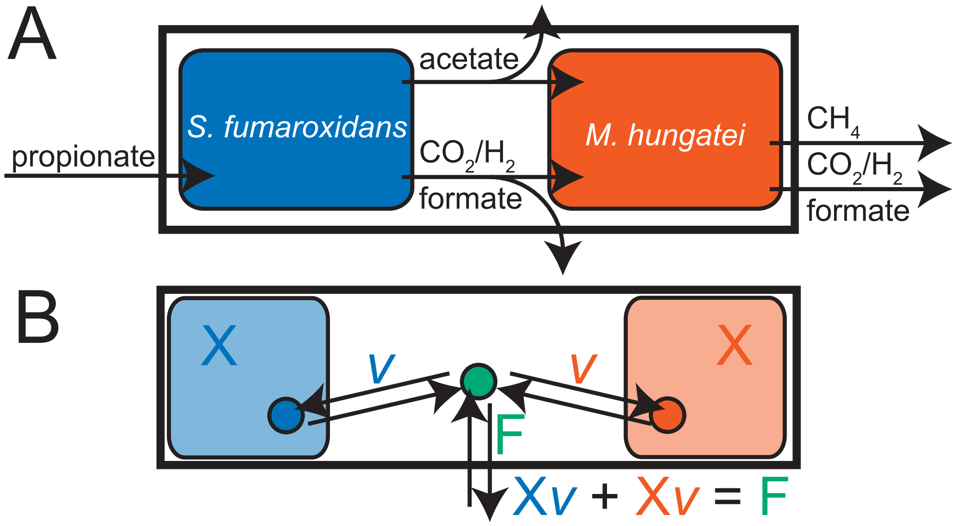

Metabolite exchange in coculture simulations. (A) Allowed carbon exchange fluxes. Propionate is fed into the system, which *S. fumaroxidans* converts into acetate, and CO_2 and H_2 or formate. These products are secreted into the media, where they can be consumed by *M. hungatei*. *M. hungatei* converts acetate to biomass, and CO_2, H_2, and formate to CH_4. *M. hungatei* can also optionally interconvert excess CO_2 and formate via a formate-hydrogen lyase. (B) Mass balance around the coculture system. In this system, both species grow at the same rate (equal to the dilution rate) and are present in the reactor at biomass concentration X. They exchange metabolites (circles) into a shared environment with flux v. The rate F represents the net flux into / out of the system, and is related to v and X for each species via the given equation. The medium components (cells and metabolites) are subject to mass balance constraints, which relate each species' biomass concentration (X), individual species uptake/secretion fluxes (v), and the net flux into or out of the reactor (F).

5 Supporting References

63. Feist AM, Scholten JCM, Palsson BØ, Brockman FJ, Ideker T (2006) Modeling methanogenesis with a genome-scale metabolic reconstruction of Methanosarcina barkeri. Mol Syst Biol 2: 2006.0004.
64. Gonnerman MC, Benedict MN, Feist AM, Metcalf WW, Price ND (2013) Genomically and biochemically accurate metabolic reconstruction of Methanosarcina barkeri Fusaro, iMG746. Biotechnol J 8: 1070–1079.
65. Satish Kumar V, Ferry JG, Maranas CD (2011) Metabolic reconstruction of the archaeon methanogen Methanosarcina Acetivorans. BMC Syst Biol 5: 28.
66. Liu Y, Beer LL, Whitman WB (2012) Methanogens: a window into ancient sulfur metabolism. Trends Microbiol 20: 251–258.
67. Pinchuk GE, Hill EA, Geydebrekht O V, De Ingeniis J, Zhang X, et al. (2010) Constraint-based model of Shewanella oneidensis MR-1 metabolism: a tool for data analysis and hypothesis generation. PLoS Comput Biol 6: e1000822.
68. Fleming RMT, Thiele I, Nasheuer HP (2009) Quantitative assignment of reaction directionality in constraint-based models of metabolism: application to Escherichia coli. Biophys Chem 145: 47–56.
69. Andersen KB, von Meyenburg K (1977) Charges of nicotinamide adenine nucleotides and adenylate energy charge as regulatory parameters of the metabolism in Escherichia coli. J Biol Chem 252: 4151–4156.
70. Van Kuijk BLM, Stams AJM (1995) Sulfate reduction by a syntrophic propionate-oxidizing bacterium. Antonie Van Leeuwenhoek 68: 293–296.
71. Anderson IJ, Ulrich LE, Lupa B, Susanti D, Porat I, et al. (2009) Genomic characterization of methanomicrobiales reveals three classes of methanogens. PLoS One 4: e5797.
72. Varma A, Palsson BØ (1994) Stoichiometric flux balance models quantitatively predict growth and metabolic by-product secretion in wild-type Escherichia coli W3110. Appl Environ Microbiol 60: 3724–3731.
73. Hamilton JJ, Reed JL (2014) Software platforms to facilitate reconstructing genome-scale metabolic networks. Environ Microbiol 16: 49–59.
74. Schut GJ, Adams MWW (2009) The iron-hydrogenase of Thermotoga maritima utilizes ferredoxin and NADH synergistically: A new perspective on anaerobic hydrogen production. J Bacteriol 191: 4451–4457.
75. Heidelberg JF, Seshadri R, Haveman SA, Hemme CL, Paulsen IT, et al. (2004) The genome sequence of the anaerobic, sulfate-reducing bacterium Desulfovibrio vulgaris Hildenborough. Nat Biotechnol 22: 554–559.
76. Biegel E, Schmidt S, González JM, Müller V (2011) Biochemistry, evolution and physiological function of the Rnf complex, a novel ion-motive electron transport complex in prokaryotes. Cell Mol Life Sci 68: 613–634.
77. Schirawski J, Unden G (1998) Menaquinone-dependent succinate dehydrogenase of bacteria catalyzes reversed electron transport driven by the proton potential. Eur J Biochem 257: 210–215.

78. Zaunmüller T, Kelly DJ, Glöckner FO, Unden G (2006) Succinate dehydrogenase functioning by a reverse redox loop mechanism and fumarate reductase in sulphate-reducing bacteria. Microbiology 152: 2443–2453.
79. Van Kuijk BLM, Schlösser E, Stams AJM (1998) Investigation of the fumarate metabolism of the syntrophic propionate- oxidizing bacterium strain MPOB. Arch Microbiol 169: 346–352.
80. Kroger A, Biel S, Simon J, Gross R, Unden G, et al. (2002) Fumarate respiration of Wolinella succinogenes: Enzymology, energetics and coupling mechanism. Biochim Biophys Acta - Bioenerg 1553: 23–38.
81. Henry CS, DeJongh M, Best AA, Frybarger PM, Linsay B, et al. (2010) High-throughput generation, optimization and analysis of genome-scale metabolic models. Nat Biotechnol 28: 977–982.
82. Mahadevan R, Bond DR, Butler JE, Coppi M V (2006) Characterization of Metabolism in the Fe(III)-Reducing Organism Geobacter sulfurreducens by Constraint-Based Modeling. Appl Environ Microbiol 72: 1558–1568.
83. Feist AM, Henry CS, Reed JL, Krummenacker M, Joyce AR, et al. (2007) A genome-scale metabolic reconstruction for Escherichia coli K-12 MG1655 that accounts for 1260 ORFs and thermodynamic information. Mol Syst Biol 3: 121.
84. Lovley DR, Giovannoni SJ, White DC, Champine JE, Phillips EJP, et al. (1993) Geobacter metallireducens gen. nov. sp. nov., a microorganism capable of coupling the complete oxidation of organic compounds to the reduction of iron and other metals. Arch Microbiol 159: 336–344.
85. Reed JL, Patel TR, Chen KH, Joyce AR, Applebee MK, et al. (2006) Systems approach to refining genome annotation. Proc Natl Acad Sci 103: 17480–17484.
86. Mahadevan R, Schilling CH (2003) The effects of alternate optimal solutions in constraint-based genome-scale metabolic models. Metab Eng 5: 264–276.
87. Mavrovouniotis ML (1990) Group contributions for estimating standard Gibbs energies of formation of biochemical compounds in aqueous solution. Biotechnol Bioeng 36: 1070–1082.
88. Jankowski MD, Henry CS, Broadbelt LJ, Hatzimanikatis V (2008) Group contribution method for thermodynamic analysis of complex metabolic networks. Biophys J 95: 1487–1499.
89. Mavrovouniotis ML (1991) Estimation of standard Gibbs energy changes of biotransformations. J Biol Chem 266: 14440–14445.
90. Schuetz R, Zamboni N, Zampieri M, Heinemann M, Sauer U (2012) Multidimensional optimality of microbial metabolism. Science 336: 601–604.
91. Shuler ML, Kargi F (2002) Bioprocess Engineering. 2nd ed. Upper Saddle River, NJ: Prentice Hall. 553 p.
92. Hamilton JJ, Reed JL (2012) Identification of Functional Differences in Metabolic Networks Using Comparative Genomics and Constraint-Based Models. PLoS One 7: e34670.
93. Ferris MC, Mangasarian OL, Wright SJ (2008) Linear Programming with Matlab. Phildelphia, PA: The Society for Industrial and Applied Mathematics and the Mathematical Programming Society. 280 p.
